# Spectral Decomposition of Chemical Semantics for Activity Cliffs‐Aware Molecular Property Prediction

**DOI:** 10.1002/advs.202517579

**Published:** 2026-02-03

**Authors:** Chaoyang Xie, Junhu Xu, Guangyi Huang, Shihang Wang, Mutian He, Xinyu Dong, Huiyang Hong, Xiaojun Yao, Qi Wang, Yuquan Li

**Affiliations:** ^1^ State Key Laboratory of Public Big Data College of Computer Science and Technology Guizhou University Guiyang China; ^2^ State Key Laboratory of Green Pesticide Key Laboratory of Green Pesticide and Agricultural Bioengineering Center for Research and Development of Fine Chemicals Ministry of Education Guizhou University Guiyang China; ^3^ Key Laboratory of Pesticide & Chemical Biology College of Chemistry Ministry of Education Central China Normal University Wuhan China; ^4^ Faculty of Applied Sciences Macao Polytechnic University Macao China

**Keywords:** activity cliffs, graph neural networks, molecular properties, molecular representations, spectral decompositions

## Abstract

Accurately predicting physicochemical and biological properties of molecules is vital for modern drug discovery, yet existing deep learning models struggle to replicate the multi‐level reasoning of chemists. Relying on single molecular graphs, they fail to capture the interplay among global scaffolds, functional groups, and pharmacophoric patterns, and often miss subtle perturbations causing “activity cliffs”. PrismNet is proposed as a spectral graph network that mimics chemical intuition through a computational prism analogy. It applies a dual‐decomposition strategy: refracting molecules into three chemical perspectives—scaffolds, functional groups, and pharmacophores—and resolving each into spectral frequencies. A dynamic learning strategy further enhances its ability to handle heterogeneous data. PrismNet achieves state‐of‐the‐art performance across 64 benchmark datasets, including 30 activity cliff datasets. Importantly, its predictions are chemically interpretable, autonomously identifying key substructures aligned with known structure‐activity relationships. This framework unifies multi‐scale semantics and spectral decomposition, enabling reliable and trustworthy in silico screening for drug discovery.

## Graphical abstract

PrismNet mimics chemical intuition by functioning as a computational prism, refracting molecular graphs into complementary semantic views and spectral frequencies. This dual‐decomposition strategy effectively captures both global topologies and subtle “activity cliff” perturbations. Achieving state‐of‐the‐art performance across 64 benchmarks, PrismNet provides an interpretable and robust solution for accelerating data‐driven drug discovery.

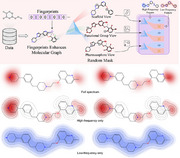

## 1 Introduction

Efficient and accurate prediction of molecular properties constitutes a pivotal step toward accelerating drug discovery, reducing experimental costs, and shortening development cycles [1, 2, 3, 4, 5, 6]. Although experimental techniques [7] are widely regarded as the gold standard, their inherent limitations, including high cost, long duration, and low throughput [8, 9], have severely hindered their scalability in the exploration of vast chemical spaces. In recent years, deep learning methodologies—particularly Graph Neural Networks (GNNs)—have emerged as transformative solutions to this problem [10, 11, 12]. Owing to their inherent compatibility with molecular graph structures, GNNs have demonstrated significant promise in surpassing traditional approaches across a wide range of molecular property prediction tasks [13].

To push the performance boundaries, recent research has coalesced around several key molecular learning paradigms. A primary direction is the integration of multimodal information with 3D geometry. For instance, some models focused on fusing data from SMILES sequences [14], 2D graphs, and 3D geometric configurations, such as PremuNet [15] and MolFuse [16]. To more accurately capture the 3D spatial structure that determines molecular properties, frameworks like SCAGE [17] enhanced conformational perception by directly integrating 3D interatomic distances into the graph attention mechanism. This focus was further refined by explicitly infusing stereoelectronic effects from natural bond orbital analysis into molecular graphs [18] or by leveraging 3D Transformer architectures to design pre‐trained models like Org‐Mol [19], which accurately predict a wide range of physical properties. Another major thrust involves innovative self‐supervised and pre‐training strategies. Researchers have introduced contrastive learning by employing line graphs as contrastive views (e.g., LEMON [20]) or by leveraging differences between 3D conformations to address domain‐shift problems [21]. Other advanced pre‐training methods include infusing hybrid noise with chemical priors and interpreting the fractional denoising task as an equivalent process of learning molecular force fields [22]; using relational learning to fuse heterogeneous modalities [23]; and applying transfer learning to address the “multi‐fidelity” data challenge in drug screening [24]. Concurrently, an emerging direction explores the integration of knowledge and reasoning from other domains, such as incorporating principles from physical chemistry (e.g., MGPhyche [25]), leveraging instructor models to mine knowledge from unlabeled data (e.g., InstructMol [26]), or integrating the capabilities of large language models (LLMs), as exemplified by the LLM4SD framework [27], which simulates the process by which human experts extract rules from literature and discover patterns from data. To address the issue of models becoming over‐optimized for specific benchmarks, the GLAM framework [28] employed an adaptive pipeline that automatically searches for optimal network architectures and hyperparameters to construct bespoke predictors for any given dataset. These technological advances have continually expanded the frontier of molecular property prediction, infusing new momentum into computational chemistry and related domains.

Nevertheless, a significant gap remains between current computational models and the deep analytical intuition employed by chemists. Rather than confining their analysis to a single atom‐level connectivity graph, chemists interpret molecules through an integrated, multi‐layered perspective encompassing scaffolds, functional groups, and pharmacophores to gain insight into core structural motifs, chemical reactivity, and biological function. This multi‐view analytical strategy is fundamental to chemical reasoning; however, most existing models rely solely on a single molecular graph representation, which limits their ability to integrate these multi‐dimensional chemical perspectives. As a result, such models often exhibit a fragmented understanding of molecular essence and struggle to capture the synergistic interplay between features across different chemical scales. Minor structural variations in molecules can lead to drastic changes in their physicochemical and biological properties; for instance, subtle modifications, such as isomerization or the fine‐tuning of substituents, may significantly alter a compound's toxicity, bioactivity, or solubility—a phenomenon widely known as “activity cliffs” [29]. A systematic study [30] involving over 60 000 models has highlighted that activity cliffs are prevalent and critical factors that significantly impact model prediction performance, yet have often been overlooked in much of the previous research on molecular property prediction. Conventional graph neural networks often suffer from excessive smoothing during message propagation [31], which tends to obscure these subtle yet critical structural distinctions and makes it difficult to model long‐range cooperative interactions between distant atoms. Moreover, datasets in drug discovery frequently suffer from sample scarcity [32, 33] and severe class imbalance, which further undermines the generalization capacity of learning algorithms [34], rendering models susceptible to overfitting or to overlooking minority‐class instances that may carry essential information [35].

To bridge this gap, we propose that an ideal model must emulate a chemist's analytical paradigm while possessing the acuity to perceive fine‐grained structural perturbations. To this end, we introduce PrismNet, a unified framework built on the central hypothesis that decomposing a molecule into its fundamental semantic and spectral components is essential for a deep understanding of its properties. PrismNet operates on this principle, functioning as a “computational prism”: first, it “refracts” the monolithic molecular graph into three complementary chemical views—scaffolds, functional groups, and pharmacophores—that embody distinct layers of chemical meaning. Second, it decomposes the structural signals within these views into low‐frequency components that encode global topology and high‐frequency components that capture local perturbations responsible for phenomena like activity cliffs. By integrating these decomposed, multi‐faceted representations, PrismNet creates a holistic and chemically rich embedding, offering a powerful, interpretable, and empirically validated solution to advance data‐driven drug discovery.

## 2 Results

### 2.1 PrismNet Acts as a Computational Prism to Decompose Molecular Information

**FIGURE 1:**
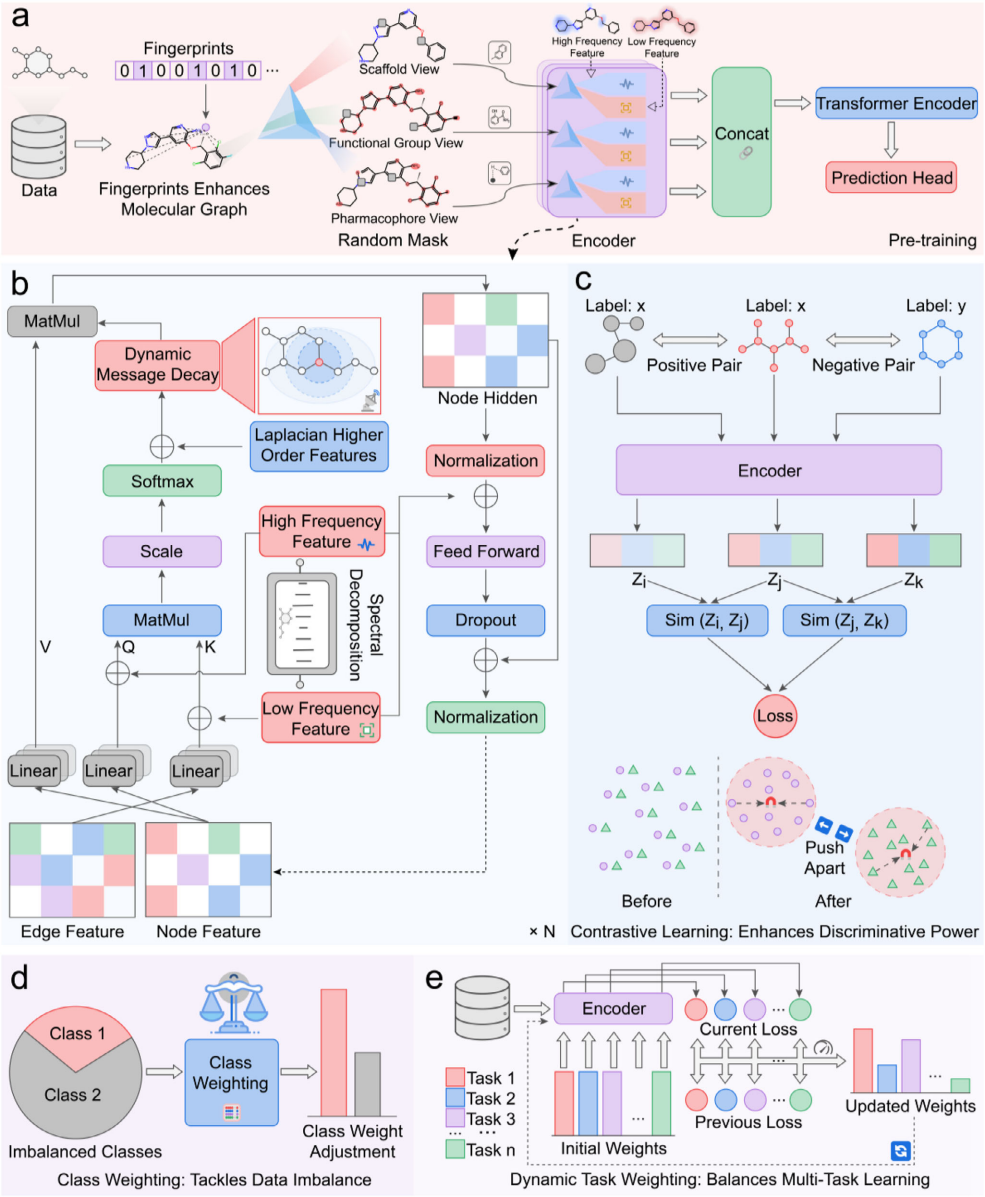
Overall architecture of the PrismNet framework. (a) PrismNet refracts a molecule into three chemically complementary semantic views: a scaffold graph, a functional group graph, and a pharmacophore graph. These are processed through a shared encoder and fused to yield comprehensive molecular embeddings. (b) The core spectral‐domain‐enhanced GNN decomposes signals into high‐frequency (local perturbations) and low‐frequency (global topology) components, which are adaptively aggregated. The framework is enhanced with supervised contrastive learning (c), class imbalance handling (d), and dynamic task weighting (e) to ensure robust and discriminative learning.

To systematically decode the complex structure‐property relationships of molecules, we developed PrismNet, a deep learning framework designed to emulate a chemist's multi‐faceted analytical approach (Figure 1). The core concept of PrismNet is to function as a computational prism, taking a singular molecular representation and decomposing it into a spectrum of more fundamental, interpretable, and informative components. This decomposition occurs along two orthogonal axes: chemical semantics and spectral frequency.

First, to capture the hierarchical nature of chemical information, PrismNet transforms a standard 2D molecular graph into three distinct, complementary views: the scaffold graph, which preserves the core topological backbone; the functional group graph, which highlights reactive centers and key substructures; and the pharmacophore graph, which abstracts features crucial for biological interactions. These views are processed by a shared graph encoder during a large‐scale pre‐training phase, forcing the model to learn a versatile “chemical language” that bridges these different levels of abstraction (Figure 1a).

Second, PrismNet introduces a spectral‐domain‐enhanced graph neural network to resolve structural signals into their constituent frequencies (Figure 1b). This is motivated by the observation that global molecular topology corresponds to low‐frequency information, while sharp, localized structural changes—such as those defining activity cliffs—manifest as high‐frequency signals. The PrismNet encoder is specifically designed to disentangle these signals, using dedicated pathways to process them before an attention mechanism adaptively reintegrates them. This spectral awareness, combined with a contrastive learning objective (Figure 1c), enables PrismNet to generate robust and chemically nuanced representations that are highly sensitive to both global structure and subtle local perturbations.

### 2.2 PrismNet Achieves State‐of‐the‐Art Accuracy Across 64 Predictive Benchmarks

To validate the predictive power of PrismNet, we conducted extensive benchmarking across a wide spectrum of 64 datasets, covering physicochemical properties, ADMET attributes, and quantum chemistry calculations from established collections like MoleculeNet [36], Therapeutics Data Commons (TDC) [37], and FS‐Mol [38]. These benchmarks were deliberately chosen to test the model's accuracy, generalization, and robustness under various conditions, including data‐scarce and multi‐task scenarios.

Across all benchmark categories, PrismNet demonstrates consistently superior or highly competitive performance. To comprehensively evaluate the model's generalization capability and practical applicability, we first assessed its performance on the MoleculeNet benchmarks, where it delivered state‐of‐the‐art results across ten out of eleven distinct tasks (Table 1). Its exceptional performance on highly imbalanced datasets like Tox21 and ClinTox highlights the effectiveness of its integrated dynamic learning strategies, confirming that PrismNet is not only accurate but also a robust and versatile tool for real‐world drug discovery challenges. We also observed that the absolute ROC‐AUC score for ToxCast is numerically lower than that of other benchmarks. This disparity is primarily attributable to the intrinsic nature of the dataset. ToxCast represents a massive multi‐task challenge comprising 617 distinct tasks that cover a wide range of distinct biological mechanisms and in vitro screening data. Consequently, the model must simultaneously learn to predict over 600 different biological outcomes, requiring it to capture highly heterogeneous structure‐activity relationships across a vast chemical space. This presents a greater optimization challenge compared to datasets with fewer or more functionally related tasks, as the model must balance potential conflicts between diverse biological signals. Furthermore, in such highly imbalanced scenarios, achieving a high ROC‐AUC is statistically more difficult because the model has very few positive samples to learn from for many of those tasks. However, it is precisely in this challenging “multi‐fidelity” landscape that PrismNet demonstrates its architectural advantage. Unlike standard models that may succumb to task dominance or class imbalance, PrismNet leverages its Dynamic Task Weighting (DTW) mechanism to adaptively balance the optimization process. By dynamically allocating focus to harder tasks, PrismNet effectively navigates this complexity, achieving highly competitive performance relative to many strong baselines even under these extreme conditions. Table S1 provides an overview of the key characteristics and statistical properties of the MoleculeNet benchmark datasets used for model evaluation.

**TABLE 1:** PrismNet demonstrates broad effectiveness and state‐of‐the‐art performance across a diverse set of classification and regression tasks within the MoleculeNet benchmark. The table systematically compares PrismNet against a suite of advanced baseline models on eleven representative datasets included in the MoleculeNet platform. These datasets span eight classification tasks and three regression tasks, covering a wide range of domains from physicochemical property prediction to bioactivity and toxicity assessment—reflecting the multifaceted nature of drug discovery. Classification tasks are evaluated using the ROC‐AUC metric, while regression tasks are assessed with RMSE; all performance scores are reported as “mean ± standard deviation”. For each dataset, an upward (↑) or downward (↓) arrow indicates whether a higher or lower score is preferable. The best results are highlighted in bold, and the second‐best results are underlined. The symbol “‐” denotes missing results in the original publications.

Datasets	BACE↑	BBBP↑	ClinTox↑	HIV↑	MUV↑	SIDER↑	Tox21↑	ToxCast↑	ESOL↓	FreeSolv↓	Lipophilicity↓
Metrics	ROC‐AUC (higher is better)								RMSE (lower is better)		
HimGNN [39]	0.856 ± 0.034	0.928 ± 0.027	0.917 ± 0.030	–	–	0.642 ± 0.023	0.807 ± 0.017	–	0.870 ± 0.154	1.921 ± 0.474	0.632 ± 0.016
TransFoxMol [40]	0.865 ± 0.005	0.925 ± 0.018	0.972 ± 0.024	–	–	0.629 ± 0.024	0.804 ± 0.010	–	0.869 ± 0.035	1.773 ± 0.368	0.633 ± 0.016
ImageMol [41]	0.839	0.739	0.851	0.797	0.825	0.660	0.773	0.653	–	–	–
KPGT [42]	0.855 ± 0.011	0.908 ± 0.010	0.946 ± 0.022	–	–	0.649 ± 0.009	0.848 ± 0.013	0.746 ± 0.002	0.803 ± 0.008	2.121 ± 0.837	0.600 ± 0.010
GEM [43]	0.856 ± 0.011	0.724 ± 0.004	0.901 ± 0.013	0.806 ± 0.009	0.817 ± 0.005	0.672 ± 0.004	0.781 ± 0.001	0.692 ± 0.004	0.798 ± 0.029	1.877 ± 0.094	0.660 ± 0.008
MGPhyche [25]	0.876 ± 0.003	0.953 ± 0.005	0.964 ± 0.014	0.820 ± 0.001	–	0.685 ± 0.019	**0.867 ± 0.004**	**0.775 ± 0.004**	0.608 ± 0.013	1.082 ± 0.045	–
PremuNet [15]	0.843 ± 0.005	0.733 ± 0.006	0.992 ± 0.002	–	–	0.626 ± 0.008	0.740 ± 0.012	–	0.851 ± 0.038	1.858 ± 0.205	0.730 ± 0.010
LEMON [20]	0.878 ± 0.014	0.737 ± 0.011	0.859 ± 0.032	0.793 ± 0.011	0.794 ± 0.043	0.643 ± 0.009	0.775 ± 0.006	0.651 ± 0.005	–	–	–
MESPool [44]	0.855 ± 0.039	0.848 ± 0.046	0.902 ± 0.065	0.756 ± 0.032	0.754 ± 0.030	0.576 ± 0.026	0.787 ± 0.019	–	1.276 ± 0.246	2.779 ± 0.762	0.708 ± 0.042
MvMRL [45]	0.878 ± 0.336	0.963 ± 0.019	0.976 ± 0.013	–	–	0.607 ± 0.023	0.794 ± 0.014	–	0.856 ± 0.088	1.770 ± 0.229	0.686 ± 0.037
KCL [46]	0.924	0.961	0.958	–	–	0.671	0.859	0.740	0.732	0.795	–
MV‐Mol [47]	0.882 ± 0.004	0.736 ± 0.002	0.956 ± 0.016	0.814 ± 0.003	0.821 ± 0.005	0.673 ± 0.000	0.803 ± 0.006	0.700 ± 0.004	0.670 ± 0.019	1.142 ± 0.258	0.566 ± 0.007
MOL‐AE [48]	0.841	0.720	0.878	0.806	0.816	0.670	–	0.696	0.830	1.448	0.607
3D PGT [49]	0.809	0.721	0.794	0.781	0.694	0.606	0.738	0.692	1.061	–	0.687
SimSGT [50]	0.843 ± 0.006	0.722 ± 0.009	0.857 ± 0.018	0.780 ± 0.019	0.814 ± 0.014	0.617 ± 0.008	0.768 ± 0.009	0.659 ± 0.008	0.917 ± 0.028	–	0.695 ± 0.012
3D INFOMAX [51]	0.794 ± 0.019	0.691 ± 0.011	0.594 ± 0.032	0.761 ± 0.013	–	0.534 ± 0.033	0.745 ± 0.007	0.644 ± 0.009	0.894 ± 0.028	2.337 ± 0.227	0.695 ± 0.012
MGSSL [52]	0.791 ± 0.009	0.697 ± 0.009	0.807 ± 0.021	0.788 ± 0.012	0.787 ± 0.015	0.618 ± 0.008	0.765 ± 0.003	0.641 ± 0.007	–	–	–
GraphFP [53]	0.805 ± 0.018	0.720 ± 0.017	0.847 ± 0.058	0.780 ± 0.015	0.754 ± 0.019	0.636 ± 0.012	0.740 ± 0.007	0.639 ± 0.009	–	–	–
S‐CGIB [54]	0.865 ± 0.008	0.888 ± 0.005	0.786 ± 0.020	0.783 ± 0.013	0.777 ± 0.012	0.640 ± 0.010	0.809 ± 0.002	0.710 ± 0.003	0.816 ± 0.019	1.648 ± 0.074	0.762 ± 0.042
MolCVG [55]	0.850 ± 0.012	0.729 ± 0.012	0.906 ± 0.017	0.792 ± 0.008	0.789 ± 0.010	0.629 ± 0.013	0.767 ± 0.007	0.644 ± 0.008	–	–	–
GRAPHMSL [56]	0.945 ± 0.007	0.943 ± 0.008	0.938 ± 0.008	0.830 ± 0.007	0.815 ± 0.037	0.673 ± 0.006	0.861 ± 0.008	0.712 ± 0.011	0.843 ± 0.094	1.601 ± 0.057	0.562 ± 0.005
InstructMol [26]	0.859 ± 0.013	0.733 ± 0.008	0.925 ± 0.021	–	–	0.674 ± 0.009	0.799 ± 0.006	0.708 ± 0.004	0.761 ± 0.043	1.604 ± 0.043	0.582 ± 0.010
PrismNet	**0.953 ± 0.001**	**0.987 ± 0.000**	**0.996 ± 0.003**	**0.856 ± 0.002**	**0.825 ± 0.003**	**0.691 ± 0.002**	**0.867 ± 0.014**	0.714 ± 0.001	**0.558 ± 0.027**	**0.654 ± 0.046**	**0.549 ± 0.017**

**FIGURE 2:**
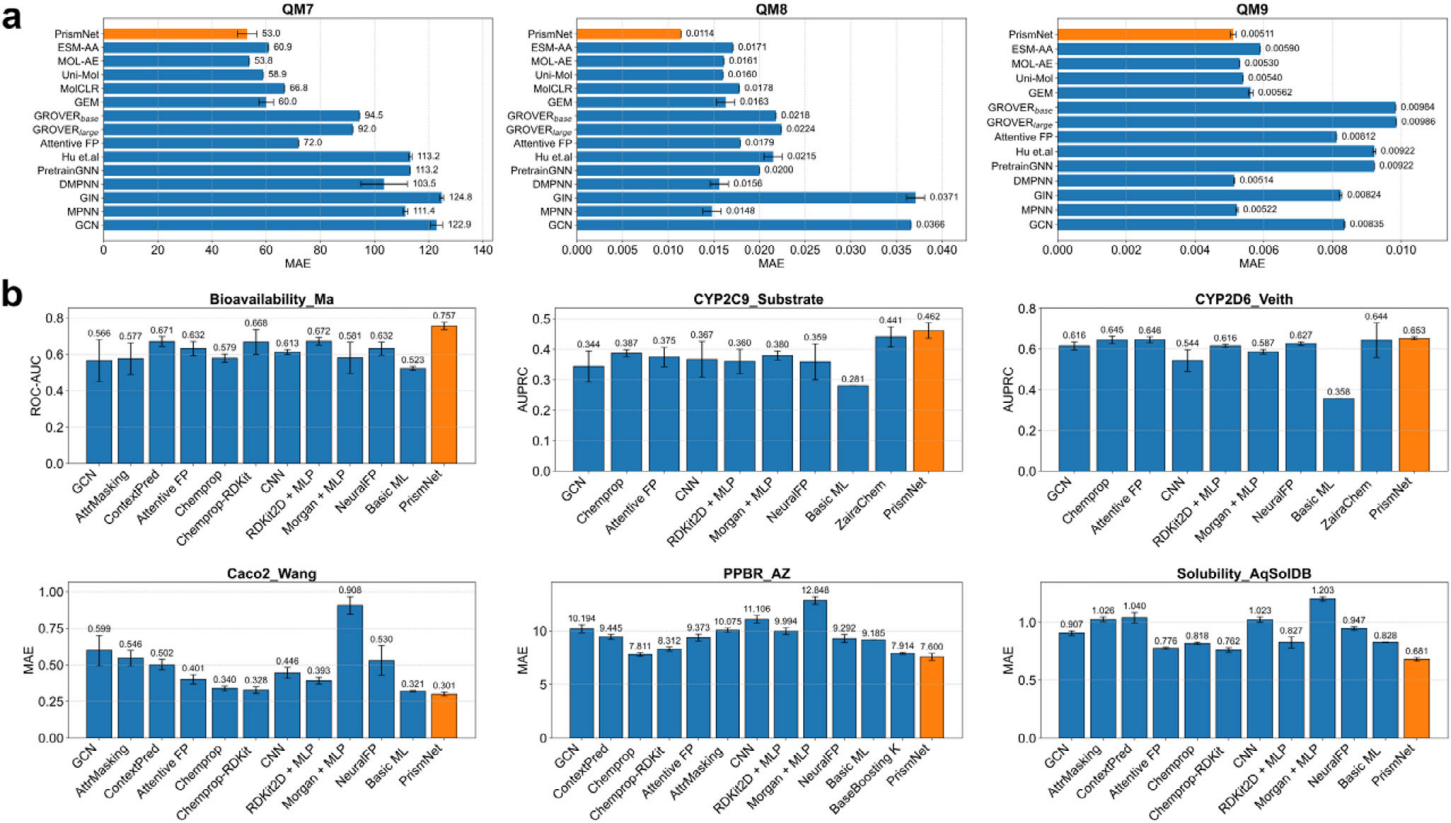
PrismNet demonstrates superior predictive performance on both the quantum chemistry datasets and TDC benchmarks. This is visually substantiated through a series of bar plots, in which PrismNet (highlighted with a red background) is directly compared against a range of competitive baseline models across two major categories of datasets, providing a comprehensive assessment of its predictive capabilities. (a) On three benchmark quantum chemistry datasets, which are exclusively regression tasks, PrismNet again outperforms all baseline models by attaining the lowest MAE scores. These results collectively confirm the model's strong capacity for accurately predicting fundamental molecular properties. (b) On six representative tasks from the TDC collection—spanning both classification tasks (evaluated using ROC‐AUC or AUPRC, where higher values indicate better performance) and regression tasks (measured by mean absolute error, MAE, where lower values are preferable)—PrismNet consistently achieves top performance. This attests to its robustness and effectiveness in modeling key pharmacological properties. Error bars indicate standard deviation across three independent runs.

Subsequently, we evaluated the model using a diverse set of representative datasets from the TDC, covering critical drug properties such as absorption, distribution, and metabolism. In this comparison, we included not only deep learning‐based methods but also traditional machine learning models like RDKit2D to ensure a more rigorous and holistic evaluation. As highlighted in the TDC benchmark study, models based on expert‐designed efficient domain features serve as highly competitive baselines for many ADMET tasks in terms of both performance and computational efficiency, in some cases even outperforming methods that rely on learned features [64]. On the TDC and quantum chemistry benchmarks, PrismNet ranks first on most tasks, outperforming a range of strong baselines (Figure 2), with detailed results reported in Tables S3–S6. For instance, it achieves the lowest mean absolute error (MAE) on all three quantum chemistry datasets (QM7 [65], QM8 [66], QM9 [67]), underscoring its ability to precisely model fundamental physical properties. Furthermore, the model's robustness was particularly evident on small, biologically relevant datasets. In the FS‐Mol benchmark, which evaluates few‐shot learning capabilities, PrismNet achieves the top rank on seven out of nine tasks, reaching an average ROC‐AUC of 0.886—a significant 2.3% relative improvement over the next best model (Table 2). Collectively, these comprehensive benchmarks establish PrismNet as a robust and broadly applicable learning method, excelling across a wide chemical and task space, from large‐scale datasets to data‐scarce, few‐shot scenarios.

**TABLE 2:** PrismNet exhibits consistently superior performance across nine small‐sized datasets from the FS‐Mol when compared to mainstream baseline methods. The table presents a detailed comparison between PrismNet and a range of competing models on these small‐scale bioactivity classification tasks, each characterized by a limited number of labeled molecular samples—a setting designed to rigorously evaluate model generalization and stability under data‐scarce conditions. All tasks are assessed using the ROC‐AUC metric, with results reported in the format of “mean ± standard deviation”. The best‐performing scores for each task are highlighted in bold, while the second‐best results are underlined for clarity.

Data ID	3215081	3888461	829401	2219110	1614450	1614408	1613998	900190	1738078	Avg
Target	–	TANK1	DHFR	IGF1R	AHR	NR2E3	JAK2	TTR	PTPN12	
Data Size	182	173	165	157	124	114	101	92	81	
TransFoxMol [40]	0.822 ± 0.130	0.859 ± 0.055	0.927 ± 0.027	0.809 ± 0.116	0.690 ± 0.098	0.940 ± 0.084	0.810 ± 0.156	**0.952 ± 0.067**	**0.958 ± 0.059**	0.863
Grover_base_ [57]	0.808 ± 0.056	0.806 ± 0.207	0.871 ± 0.055	0.667 ± 0.139	0.676 ± 0.305	0.826 ± 0.104	0.794 ± 0.157	0.882 ± 0.130	0.867 ± 0.109	0.800
AttentiveFP [58]	0.862 ± 0.032	0.793 ± 0.140	0.852 ± 0.027	0.637 ± 0.100	0.654 ± 0.157	0.812 ± 0.077	0.762 ± 0.039	0.800 ± 0.163	0.936 ± 0.051	0.790
GEM [43]	0.792 ± 0.118	0.722 ± 0.080	0.867 ± 0.040	0.664 ± 0.100	0.606 ± 0.014	0.802 ± 0.044	0.762 ± 0.103	0.839 ± 0.190	0.872 ± 0.102	0.770
MAT [59]	0.771 ± 0.132	0.708 ± 0.120	0.882 ± 0.058	0.635 ± 0.228	0.597 ± 0.066	0.674 ± 0.075	0.730 ± 0.098	0.808 ± 0.059	0.750 ± 0.204	0.728
PretrainGNN [60]	0.776 ± 0.164	0.686 ± 0.017	0.812 ± 0.070	0.647 ± 0.135	0.555 ± 0.216	0.640 ± 0.105	0.492 ± 0.136	0.839 ± 0.105	0.911 ± 0.063	0.707
DMPNN [61]	0.719 ± 0.116	0.715 ± 0.274	0.830 ± 0.069	0.656 ± 0.043	0.522 ± 0.027	0.586 ± 0.176	0.635 ± 0.059	0.489 ± 0.350	0.794 ± 0.152	0.661
GraphMVP [62]	0.820 ± 0.095	0.682 ± 0.075	0.837 ± 0.080	0.597 ± 0.188	0.454 ± 0.160	0.418 ± 0.096	0.508 ± 0.022	0.883 ± 0.103	0.579 ± 0.252	0.642
MolCLR [63]	0.768 ± 0.082	0.716 ± 0.094	0.867 ± 0.072	0.634 ± 0.158	0.538 ± 0.118	0.578 ± 0.088	0.333 ± 0.189	0.638 ± 0.068	0.367 ± 0.262	0.604
PrismNet	**0.900 ± 0.021**	**0.892 ± 0.029**	**0.948 ± 0.031**	**0.843 ± 0.090**	**0.711 ± 0.057**	**0.948 ± 0.027**	**0.843 ± 0.064**	0.950 ± 0.101	0.938 ± 0.073	**0.886**

### 2.3 PrismNet's Representations Are Chemically Interpretable and Trustworthy

**FIGURE 3:**
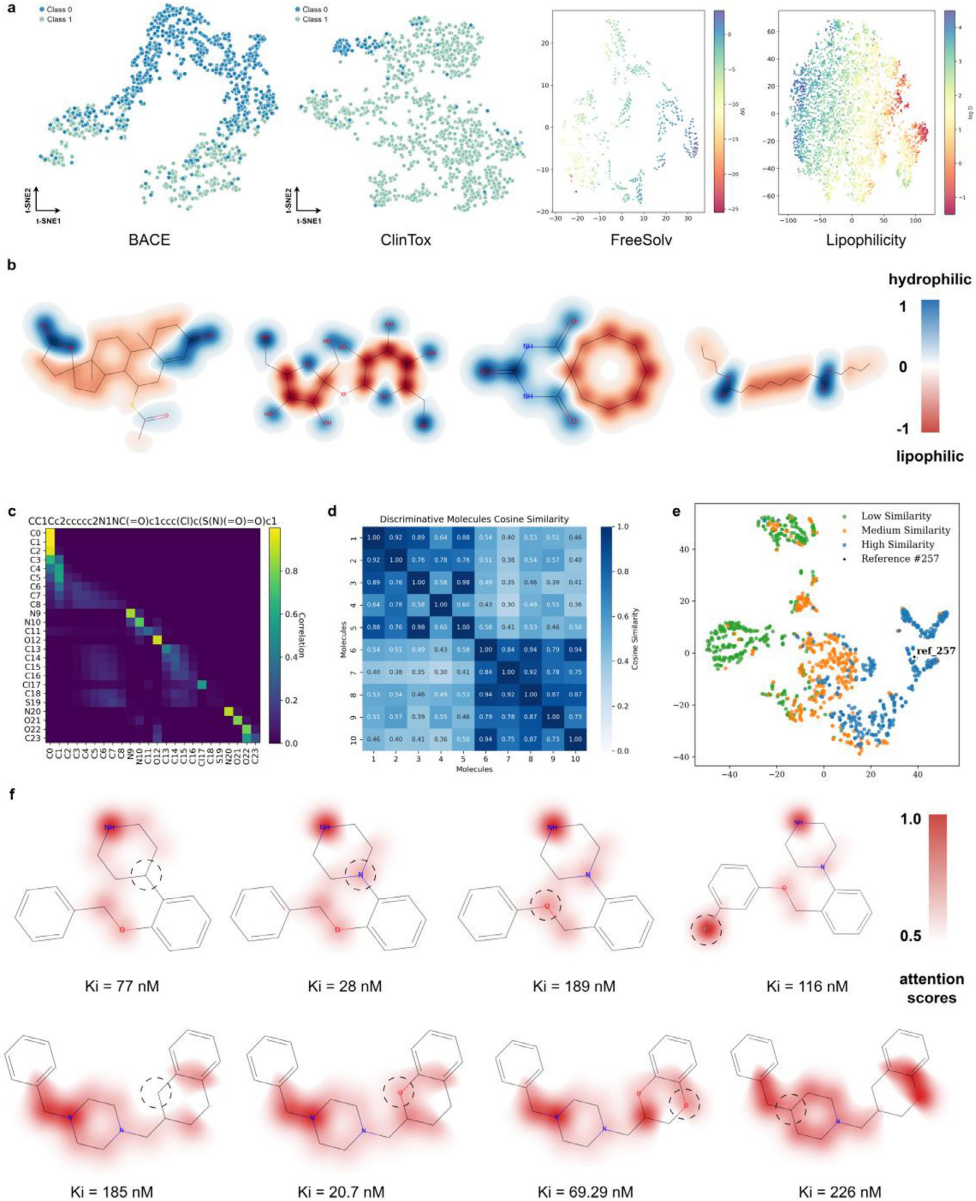
The molecular representations learned by PrismNet are not only chemically plausible but also highly interpretable, with decision logic that aligns closely with established chemical principles and structural similarity patterns. (a) The embeddings produced by PrismNet exhibit strong class separability and structure‐property continuity in the learned feature space. (b) Node‐level interpretation cases further illustrate how PrismNet identifies substructures critical to solubility prediction. Here, each atom is color‐coded according to its contribution weight: blue regions indicate hydrophilic groups (weight close to 1), while red regions highlight lipophilic moieties (weight close to ‐1). (c) The intra‐molecular atom correlation heatmap captures the pairwise dependencies among atoms as learned by the model, with color intensity reflecting the magnitude of inter‐atomic relevance. This visualization reveals how PrismNet implicitly partitions molecules into functional subregions. (d) The molecular representations learned by PrismNet exhibit high intra‐class compactness and inter‐class separability. A cosine similarity heatmap of feature vectors from ten randomly selected molecules in the HIV dataset shows distinct clustering, with molecules 1–5 belonging to the positive class and molecules 6–10 to the negative class. (e) PrismNet's learned representations also preserve structural similarity among molecules. A t‐SNE projection of ESOL embeddings demonstrates this property: a randomly selected molecule (Reference #257) is marked with a black pentagram, while all other molecules are color‐coded by their Tanimoto similarity to the reference based on Morgan fingerprints. The molecules are divided into three groups (low, medium, and high similarity) based on the 33rd and 66th percentiles of the Tanimoto similarity distribution—green indicating low similarity, orange medium, and blue high. (f) Visualization of the PrismNet model's attention on activity cliff pairs. The red shading indicates atom‐level attention scores, where a deeper color represents a greater contribution of the atom to the activity prediction.

A critical requirement for any model in drug discovery is that its decision‐making process be transparent and aligned with established chemical intuition. We investigated whether PrismNet's internal representations are chemically meaningful. By projecting the learned embeddings of molecules onto a 2D space using t‐SNE [68], we observed that PrismNet autonomously organizes molecules according to their properties without explicit instruction (Figure 3a). For classification tasks like BACE and ClinTox, molecules with different labels form distinct and compact clusters. For regression tasks like ESOL (solubility), the embeddings form a smooth gradient that directly corresponds to the continuous property values, demonstrating that the learned space is well‐structured and meaningful.

To further probe the model's chemical reasoning, we visualized the atomic contribution weights for solubility prediction (Figure 3b,c). PrismNet correctly assigns positive importance (hydrophilic, blue) to polar groups like hydroxyls and negative importance (lipophilic, red) to nonpolar moieties like aromatic rings, perfectly mirroring a chemist's qualitative assessment. This ability to identify and prioritize relevant chemical substructures demonstrates a deep, learned understanding of structure‐property principles.

We assessed whether the learned representations preserve both functional and structural similarity. A similarity [69] heatmap on the HIV dataset shows high intra‐class similarity and low inter‐class similarity, confirming that PrismNet effectively groups molecules by their biological activity (Figure 3d). Moreover, an analysis on the ESOL dataset reveals that molecules structurally similar to a reference compound (as measured by Tanimoto similarity) [70] are clustered closely together in PrismNet's embedding space (Figure 3e). Together, these results provide compelling evidence that PrismNet learns representations that are not just predictive, but also interpretable and chemically trustworthy.

**FIGURE 4:**
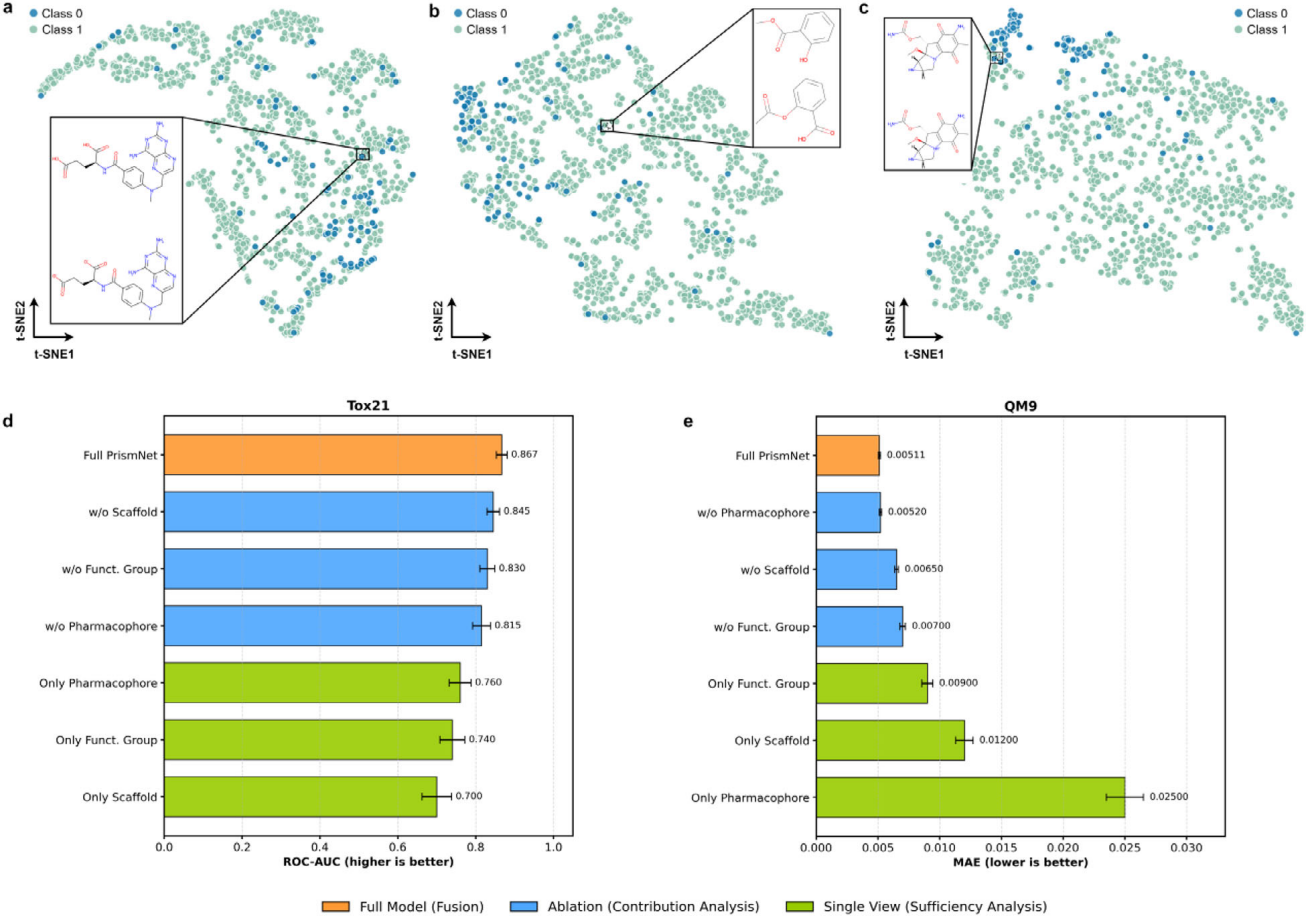
Qualitative and quantitative validation of the multi‐view complementarity strategy. (a)‐(c), t‐SNE visualization of multi‐view embeddings reveals PrismNet's complementary decomposition strategy. These scatter plots show the results of t‐SNE projection into two dimensions of molecular embeddings generated from the ClinTox dataset, encoded by a single view: (a) scaffold, (b) functional group, and (c) pharmacophore. Each point represents a molecule, colored according to its true toxicity class (Class 0 or Class 1). (d,e), Quantitative contribution analysis of chemical views across two representative tasks. Error bars represent the standard deviation across three independent runs.

To visually validate the effectiveness and complementarity of the PrismNet multi‐view decomposition strategy, we conducted a series of ablation experiments and visualized the molecular embeddings. As shown in Figure 4, we performed t‐SNE projection into two dimensions on molecular embeddings generated solely by the scaffold view, functional group view, and pharmacophore view, as well as the embeddings from the complete PrismNet model (Figure 3a), and colored them according to their true toxicity classes. The visualizations clearly show the limitations of every single view and their complementarity. In the embedding space generated using only the scaffold view, molecular points of different classes are intermingled without forming clear boundaries or clusters. This indicates that while the core topological scaffold information of a molecule is important, it is insufficient to distinguish the critical local structures that drive toxicity differences. When using the functional group view, the class separation becomes more distinct, but the overlap between classes remains significant. This suggests that isolated functional group information cannot fully capture complex structure‐activity relationships. The pharmacophore view exhibits a different clustering pattern from the previous two, highlighting features potentially related to interactions with biological targets, but also does not achieve clear class separation.

To further quantitatively validate the contribution of each view across different chemical domains, we conducted systematic ablation and single‐view sufficiency experiments on two representative datasets: Tox21 (biological toxicity classification) and QM9 (quantum chemical regression). As shown in Figure 4d,e, the importance of each view varies significantly depending on the task nature, yet the multi‐view fusion consistently yields the best performance. On the Tox21 dataset, the Pharmacophore view emerged as the most critical individual component, with the “Only Pharmacophore” model achieving an ROC‐AUC of 0.760, significantly outperforming the Scaffold‐only baseline (0.700). Conversely, removing the pharmacophore view resulted in the largest performance drop, underscoring the pivotal role of pharmacophoric features in determining biological interactions and toxicity. In contrast, for the QM9 dataset, which predicts fundamental physical properties, the Scaffold and Functional Group views proved dominant. The “Only Pharmacophore” model exhibited a substantially higher error compared to the “Only Scaffold” model, indicating that abstract pharmacophoric features alone are insufficient to capture precise electronic states. Notably, even in this scaffold‐dominated task, the Full PrismNet model outperformed the “w/o Pharmacophore” variant, demonstrating that integrating multi‐scale chemical semantics provides complementary information necessary for achieving state‐of‐the‐art accuracy.

These results strongly demonstrate that chemical information of molecules has a hierarchical nature, and a single view can only capture partial information. Therefore, fusing multiple chemical semantic views is crucial for building a comprehensive and trustworthy molecular representation. This not only explains the reasons behind PrismNet's superior performance but also provides intuitive visual evidence for understanding how the model simulates a chemist's multi‐view analytical thinking.

### 2.4 Spectral Decomposition Is Critical for Predicting Activity Cliffs

A central hypothesis of our work is that the key to accurately modeling molecular properties—especially for structurally sensitive cases like activity cliffs—lies in the ability to decouple and integrate information from different structural scales. PrismNet's spectral decomposition module is designed precisely for this purpose, separating localized, high‐frequency signals from global, low‐frequency patterns.

We performed a rigorous experimental evaluation on the challenging task of activity cliff prediction. For this work, we used the 30 activity cliff datasets introduced in a previous study [29, 71]. To ensure a fair comparison, we followed the dataset partitioning scheme provided by the original authors and compared the PrismNet model against a series of baseline methods, using the root mean square error to quantify the predictive performance of the models. As shown in Table 3, the experimental results demonstrate the superiority of our model, with PrismNet outperforming the other compared models on 29 of the 30 datasets. This remarkable performance strongly suggests that the spectral decomposition mechanism is adept at capturing the subtle structural perturbations that define activity cliffs.

**TABLE 3:** Performance comparison of PrismNet on activity cliff prediction tasks. This table provides a detailed comparison of the predictive performance of PrismNet against various baseline models on 30 activity cliff datasets. The evaluation metric is the Root Mean Square Error (RMSE), for which lower values indicate superior performance. In each row, the best result is highlighted in bold, and the second‐best result is underlined.

Datasets	Attentive FP [58]	CNN [72]	GAT [73]	GCN [74]	MPNN [75]	ImageMol [41]	GEM [43]	PrismNet
Metrics	RMSE (lower is better)							
CHEMBL1862_Ki	1.347	1.049	0.987	0.942	0.948	0.773	0.743	**0.709**
CHEMBL1871_Ki	1.143	0.810	0.798	0.769	1.058	0.758	0.715	**0.665**
CHEMBL2034_Ki	0.931	0.798	0.809	0.810	0.905	0.737	0.763	**0.683**
CHEMBL2047_EC50	0.970	0.776	0.840	0.797	1.030	**0.629**	0.674	0.641
CHEMBL204_Ki	1.553	1.131	1.138	1.056	1.458	0.854	0.837	**0.798**
CHEMBL2147_Ki	1.906	0.925	0.966	0.840	1.025	0.810	0.801	**0.742**
CHEMBL214_Ki	1.083	0.931	1.051	1.007	1.183	0.767	0.775	**0.758**
CHEMBL218_EC50	1.046	0.958	0.957	0.928	1.053	0.755	0.795	**0.679**
CHEMBL219_Ki	0.952	0.977	0.979	1.026	0.903	0.837	0.889	**0.820**
CHEMBL228_Ki	1.192	0.965	1.026	0.958	1.000	0.786	0.767	**0.765**
CHEMBL231_Ki	1.262	1.008	0.991	0.878	1.305	0.777	0.726	**0.626**
CHEMBL233_Ki	1.211	1.073	1.066	1.056	1.074	0.817	0.804	**0.785**
CHEMBL234_Ki	0.885	0.898	0.950	0.934	0.959	0.725	0.752	**0.717**
CHEMBL235_EC50	1.202	0.893	0.869	0.901	1.058	0.693	0.717	**0.669**
CHEMBL236_Ki	1.370	1.018	1.002	0.942	1.364	0.836	0.805	**0.771**
CHEMBL237_EC50	1.361	1.061	1.103	1.132	1.402	0.922	0.926	**0.829**
CHEMBL237_Ki	1.310	1.040	1.085	1.112	1.053	0.820	0.761	**0.754**
CHEMBL238_Ki	1.216	0.917	0.928	0.937	1.142	0.743	0.697	**0.606**
CHEMBL239_EC50	1.361	0.910	0.902	0.906	1.288	0.748	0.774	**0.739**
CHEMBL244_Ki	1.706	1.095	1.088	1.075	1.660	0.864	0.894	**0.811**
CHEMBL262_Ki	1.116	0.948	0.994	0.934	1.021	0.837	0.830	**0.748**
CHEMBL264_Ki	1.102	0.890	0.896	0.855	1.082	0.691	0.661	**0.642**
CHEMBL2835_Ki	0.747	0.560	0.555	0.505	0.668	0.468	0.466	**0.403**
CHEMBL287_Ki	1.149	0.891	0.947	0.886	0.927	0.809	0.793	**0.774**
CHEMBL2971_Ki	1.091	0.831	0.803	0.781	0.973	0.740	0.708	**0.642**
CHEMBL3979_EC50	1.080	0.907	0.923	0.812	1.183	0.851	0.817	**0.704**
CHEMBL4005_Ki	1.059	0.838	0.861	0.875	0.998	0.722	0.650	**0.611**
CHEMBL4203_Ki	1.062	1.013	1.004	0.975	1.056	1.003	0.897	**0.831**
CHEMBL4616_EC50	0.947	0.819	0.873	0.867	0.935	0.738	0.715	**0.632**
CHEMBL4792_Ki	1.233	0.967	1.004	0.923	1.122	0.801	0.825	**0.763**

To understand how PrismNet achieves this, we conducted an interpretive analysis of activity cliff pairs using attention mechanisms, as illustrated in Figure 3f and Figure S6. The figure displays several molecular pairs that are highly similar in structure but exhibit significant differences in biological activity (represented by the inhibition constant, Ki). Each pair in a set has only minor differences in chemical structure (indicated by dashed circles), reflecting the characteristics of structure‐activity cliffs. We can observe that PrismNet accurately identifies the key structural regions, such as substituents or functional groups, that are responsible for the changes in activity. The model's areas of focus are highly consistent with the locations in the molecular structure that trigger the drastic change in activity, which demonstrates that the high‐ and low‐frequency signal modeling has excellent sensitivity when handling structure‐activity relationships.

**FIGURE 5:**
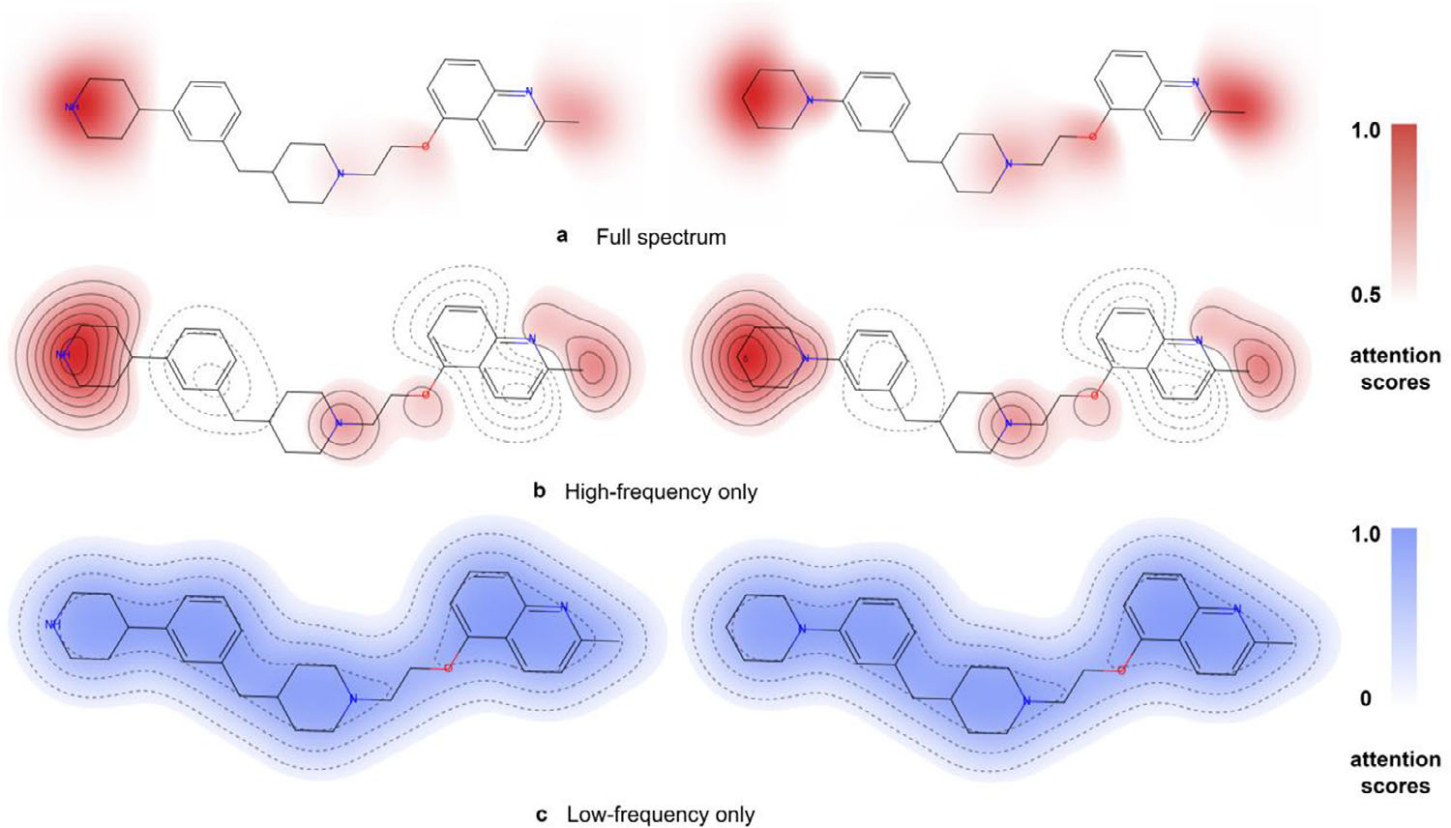
Visualization of spectral decomposition on an activity cliff pair from the CHEMBL214_Ki dataset. (a) The full‐spectrum attention weights are projected onto the molecular structures. (b) The high‐frequency component visualization. Attention scores are mapped using a red color scale (thresholded range [0.5, 1.0]) superimposed with contour lines. The dense clustering of contours reveals sharp attention gradients, effectively emphasizing sharp, localized signals associated with structural variations. (c) The low‐frequency component visualization. Attention scores are mapped using a blue color scale (full range [0, 1.0]) overlaid with sparse, widely spaced contours to capture the smooth, continuous global topology. This contrast visualizes how PrismNet uses high‐frequency signals to pinpoint local mutation sites while retaining global scaffold context via low‐frequency channels.

To further elucidate the mechanism by which PrismNet captures subtle structural perturbations in activity cliffs, we conducted a granular interpretability analysis on a representative molecular pair from the CHEMBL214_Ki dataset. As illustrated in Figure 5, the model's spectral decomposition module effectively disentangles the learned representations into frequency‐specific components, each playing a distinct semantic role. Specifically, the high‐frequency component (Figure 5b) demonstrates a high sensitivity to local structural discontinuities, evidenced by the dense clustering of contour lines that reveal sharp attention gradients. By applying a focused attention threshold, we observed that the high‐frequency signals prominently highlight the local structural perturbations corresponding to the bioactivity‐determining substitution site, thereby precisely identifying the critical determinants of bioactivity. This aligns with the graph signal processing theory that high eigenvalues correspond to rapidly varying signals, such as specific functional group modifications. In contrast, the low‐frequency component (Figure 5c) exhibits a spatially diffuse attention pattern distributed across the entire molecular backbone. Visualized using a full‐range scale overlaid with sparse, widely spaced contours, these signals encapsulate the global topological continuity and scaffold identity, which remain invariant between the paired molecules. The effective disentanglement of these components corroborates our hypothesis: PrismNet does not merely rely on global structural similarity but actively decomposes chemical semantics, leveraging high‐frequency signals to capture cliff‐inducing perturbations while utilizing low‐frequency signals to represent the conserved scaffold context.

**FIGURE 6:**
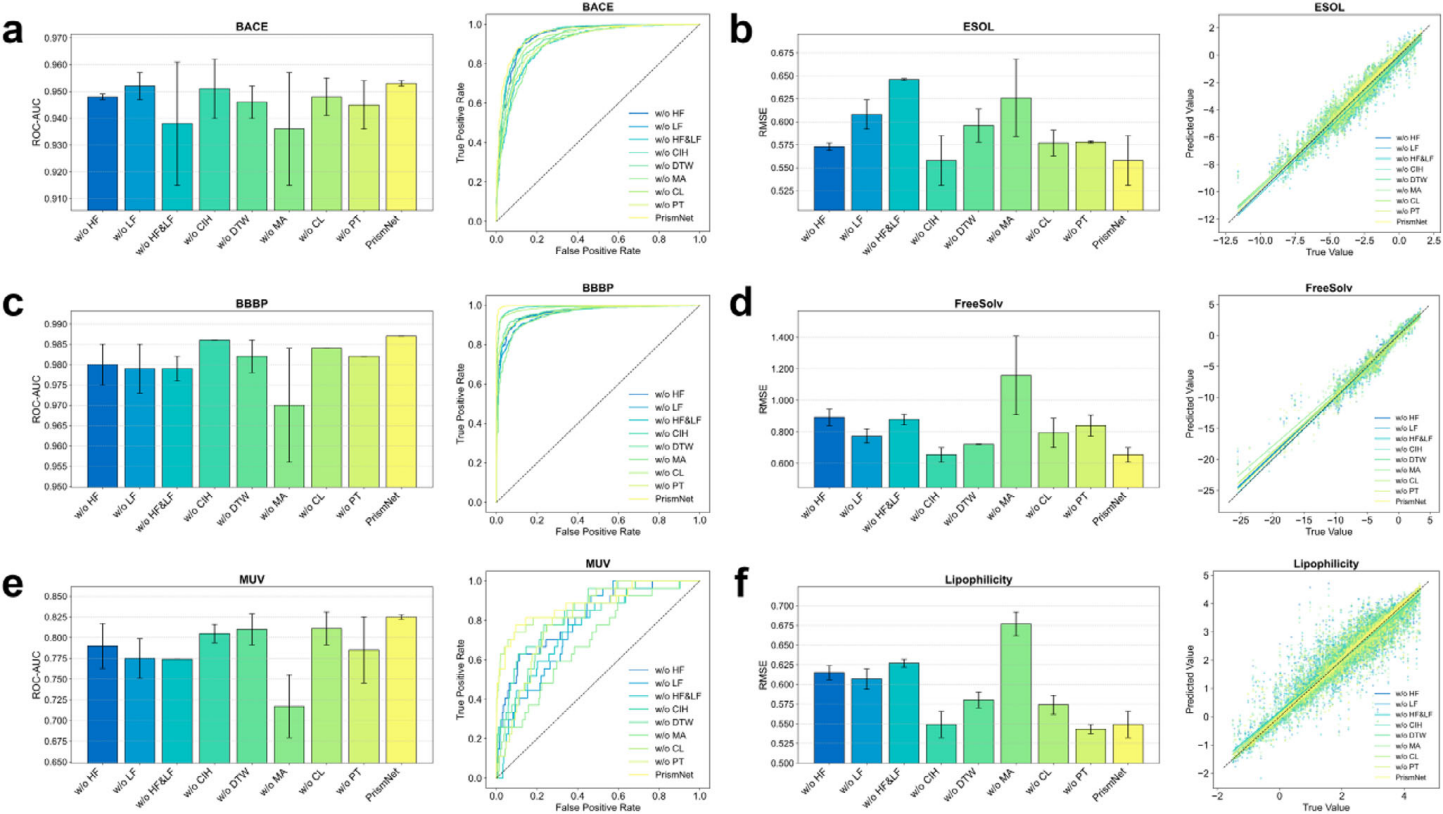
The ablation study systematically verifies the necessity and effectiveness of PrismNet's core components. The figure presents ablation results on a series of classification and regression datasets, aiming to assess the individual contribution of each module to the overall performance. Variants of the PrismNet model were constructed by selectively removing specific components while keeping all other parameters unchanged for fair comparison. Results are shown for three classification tasks and three regression tasks. Each task includes a bar chart comparing performance metrics (ROC‐AUC for classification, RMSE for regression) across model variants, accompanied by visualizations—ROC curves for classification and scatter plots of predicted vs. true values for regression—to further illustrate performance differences. The ablation variants are defined as follows: without high‐frequency information (w/o HF), without low‐frequency information (w/o LF), without both high‐ and low‐frequency information (w/o HF&LF), without class imbalance handling (w/o CIH), without dynamic task weighting (w/o DTW), without message attenuation (w/o MA), without contrastive learning (w/o CL), and without pre‐training (w/o PT). Error bars indicate standard deviation across three independent runs.

To validate the criticality of this mechanism, we conducted a series of systematic ablation studies. The results, summarized in Figure 6, provide compelling evidence for our hypothesis. When the high‐frequency information filter was removed (w/o HF), model performance degraded significantly across both classification (e.g., BACE) and regression (e.g., ESOL) tasks. This confirms the importance of capturing sharp, local structural variations for accurate prediction. Conversely, removing the low‐frequency filter (w/o LF) also impairs performance, indicating that a holistic understanding of the molecule's global topology is equally essential. Unsurprisingly, removing both spectral components (w/o HF&LF) leads to the most substantial performance drop, demonstrating that these two information channels are complementary and synergistically contribute to PrismNet's predictive power.

Furthermore, we ablated the multi‐view pre‐training strategy (w/o PT), which constitutes the other facet of PrismNet's “decomposition” approach. Disabling this module also leads to a consistent decrease in performance. This finding underscores that decomposing the input into different semantic views (scaffold, functional group, pharmacophore) is as crucial as decomposing the graph signal into different frequencies. It is the powerful combination of these two orthogonal decomposition strategies—one semantic, one spectral—that allows PrismNet to build such robust and sensitive molecular representations.

### 2.5 Dynamic Learning Strategies Confer Robustness on Imbalanced Datasets

Real‐world datasets in drug discovery are notoriously heterogeneous, often suffering from severe class imbalance and substantial variance in difficulty across different predictive tasks (Figure S1). Standard models trained with uniform objectives tend to be biased toward majority classes and easier tasks, leading to poor generalization. To address this, PrismNet incorporates a dynamic learning strategy designed to enhance its robustness and performance in these challenging, true‐to‐life scenarios.

This strategy integrates two key mechanisms: class imbalance handling (CIH) and dynamic task weighting (DTW), as depicted in Figure 1d,e. The CIH component automatically calculates and applies class‐specific weights during loss computation, forcing the model to pay more attention to rare but often critical minority‐class samples (e.g., toxic compounds or active inhibitors). Concurrently, for multi‐task learning scenarios, the DTW mechanism adaptively adjusts the weight of each task based on its learning progress. This allows the model to dynamically allocate more focus to more difficult or slower‐converging tasks, ensuring a balanced optimization process across the board.

The efficacy of this dynamic strategy is demonstrated by ablation studies, as shown in Figure 6 and Figure S3. On highly imbalanced datasets such as MUV, removing the CIH module results in a marked drop in predictive performance, validating its role in learning from sparse positive signals. Similarly, removing the DTW module in multi‐task settings leads to a decline in average performance, confirming its value in navigating task heterogeneity. Ablating the supervised contrastive learning loss [76] (w/o CL), which encourages intra‐class compactness and inter‐class separation, further diminishes performance, as it is a key element in learning discriminative features, especially for challenging minority classes. This integrated dynamic learning approach is therefore critical to PrismNet's robustness and its successful application to complex, real‐world data.

## 3 Conclusion

By emulating a chemist's reasoning, PrismNet advances molecular property prediction through a dual‐decomposition strategy. It first refracts molecules into fundamental chemical perspectives—scaffolds, functional groups, and pharmacophores—and then resolves these views into distinct spectral frequencies. This hierarchical analysis, combined with dynamic learning strategies, allows PrismNet not only to achieve state‐of‐the‐art accuracy but also to generate chemically interpretable predictions. During the pre‐training phase, the model learns generalizable chemical rules from large‐scale data, while in the fine‐tuning phase, it effectively addresses common challenges in drug discovery, such as data sparsity and imbalance, through dynamic task balancing and contrastive learning strategies. The core strength of PrismNet lies not only in its predictive accuracy but also in its ability to emulate the analytical reasoning of chemists, thereby ensuring strong chemical interpretability throughout the prediction process.

The experimental results demonstrate not only the superior predictive accuracy of PrismNet across various benchmark datasets but also—more importantly—its ability to autonomously learn and focus on structural features consistent with chemical intuition, as validated by interpretability analyses. For instance, the attention mechanism reliably highlights polar functional groups that influence solubility, while t‐SNE embeddings distinctly partition molecular clusters according to biological activity. This indicates that PrismNet is not only a high‐performance predictive tool but also a promising framework for chemical knowledge discovery that can uncover valuable structure‐activity relationships from data. Moreover, we hypothesize that different chemical views inherently carry spectral signals at distinct scales. PrismNet leverages this property by first acquiring chemically and spectrally diverse information streams through multi‐view inputs, and then decomposing these signals via the spectral enhancement module. The effectiveness of this mechanism is further evidenced by ablation results: removing multi‐view pre‐training or selectively eliminating high‐ or low‐frequency signal paths consistently results in degraded performance. This design, representing a deep integration of chemical intuition and spectral‐domain analysis, may be one of the key factors behind PrismNet's exceptional performance.

Despite its promising results, PrismNet has several limitations. From a chemical perspective, the current version of PrismNet is primarily based on 2D molecular graphs. Although spectral enhancement may indirectly capture features related to 3D structure, it does not explicitly model molecular conformations, chirality, or dynamic flexibility—factors that are critical for certain bioactivity predictions, such as precise enzyme‐inhibitor interactions. Recent studies, such as Uni‐Mol+, have demonstrated that explicitly modeling the 3D equilibrium conformations of molecules can significantly improve the accuracy of quantum chemical property prediction, highlighting the critical role of 3D geometry in molecular representation [77]. Additionally, on complex datasets like ToxCast, which involve a wide range of distinct biological mechanisms, PrismNet's performance still leaves room for improvement. This suggests that a general‐purpose model may struggle to fully capture all target‐specific interaction patterns, highlighting the potential need for future enhancements incorporating target structure information or biological pathway knowledge. Moreover, although the pre‐training dataset is large, its chemical space coverage may limit the model's generalization ability to novel scaffolds or rare chemical elements.

Our future work will extend along several directions. At the model level, we aim to integrate explicit 3D structural information and molecular dynamics simulation data into the PrismNet framework, with the goal of more accurately predicting conformation‐sensitive properties. On the application front, we plan to explore the deployment of PrismNet in broader drug discovery pipelines, including efficient virtual screening, lead compound optimization, retrosynthetic analysis, and even novel material design. In terms of interpretability, we intend to develop more advanced analytical tools that not only highlight key atoms or functional groups but also reveal their complex cooperative or antagonistic interaction patterns, thereby offering more actionable insights for molecular design. For specific disease domains or target families, we will leverage transfer learning and domain adaptation techniques to construct highly customized versions of PrismNet, aiming to enhance its utility in the context of precision medicine.

## 4 Methods

### 4.1 Pre‐Training Strategy for Multi‐View Representation Learning

A powerful predictive model must extend beyond accuracy—it must also be trustworthy, with decision‐making logic that aligns with established chemical principles. To enable the model to emulate the analytical reasoning of chemists—examining molecules through multiple layers of chemical abstraction and thereby acquiring a universal “chemical language” and a set of structure‐property heuristics—we devised a multi‐view pre‐training strategy. The strategy applies three chemically inspired transformations to the original molecular graph G, resulting in three complementary structural representations: the scaffold graph. $G_{s}$, the functional group graph $G_{f}$, and the pharmacophore graph $G_{p}$. The scaffold graph captures the core molecular scaffold and global topology, which are essential for understanding the overall geometry and structural backbone of the molecule. The functional group graph highlights chemically reactive substructures that govern intermolecular interactions and largely determine the molecule's physicochemical properties. The pharmacophore graph abstracts the spatial and chemical features essential for molecular recognition—such as hydrogen bond donors/acceptors and hydrophobic centers—and plays a pivotal role in predicting biological activity. By jointly modeling these semantically distinct graph views, the framework achieves a more comprehensive and chemically informed understanding of molecular structure.

During the pre‐training phase, we formulated a multi‐task node prediction objective to encourage the model to learn generalizable representations of molecular graphs. Chemically, this node‐level prediction task compels the model to reconstruct the attributes of a central atom—or of a global information node—based solely on its surrounding chemical environment, namely the neighboring atoms and the bonds that connect them. This design not only enables the model to capture intrinsic atomic properties, but more critically, it facilitates the understanding of interatomic interactions and how such localized structural dependencies collectively shape the global chemical behavior of the molecule.

Given a molecular graph G=(V,E), where V denotes the set of atom nodes, and E denotes the set of edges representing chemical bonds (with detailed feature definitions provided in Table S2), we first generate the three heterogeneous graph views. The algorithmic details of this multi‐view generation process are described in the “Multi‐view generation algorithm” section of the Supplementary Information. Each view is encoded by a shared‐parameter graph neural network encoder *f*_θ_(·), yielding node‐level hidden representations:

(1) $$\begin{array}{c} H_{s}=f_{\theta }\;\left(G_{s}\right),\\ \;H_{f}=f_{\theta }\;\left(G_{f}\right),\\ \;H_{p}=f_{\theta }\;\left(G_{p}\right) \end{array}$$

The three node representation sets are concatenated along the feature dimension to form the fused features:

(2) $$H_{fusion}=\left[H_{s};H_{f};H_{p}\right]\;$$

The fused features *H_fusion_* are then fed into a Transformer module, where each node functions as a token, and the global information node is included as a special token. The Transformer output *H_out_* is used for node‐level value prediction. For each atomic node v∈V, the model predicts ${\hat{h}}_{v}$ and computes the squared error loss against the ground‐truth value *h_v_*. Likewise, for the global information node *k*, the model predicts ${\hat{h}}_{k}$ and compares it with the ground‐truth value *h_k_*. The pre‐training objective is to minimize the total loss across all nodes:

(3) $$L_{pretrain}=\sum_{v\in V}∥{\hat{h}}_{v}-h_{v}{∥}^{2}+∥{\hat{h}}_{k}-h_{k}{∥}^{2}\;$$

### 4.2 Graph Neural Network with Frequency Domain Enhancement

Spectral‐domain analysis is critical for identifying molecules exhibiting activity cliffs. The ability to simultaneously capture local structural perturbations (high‐frequency information) and global topological patterns (low‐frequency information) is a key mechanism for achieving accurate molecular property prediction. Molecular properties are often highly sensitive to subtle structural modifications—a phenomenon known as activity cliffs—which poses a significant challenge for computational models. We hypothesize that this difficulty stems from the model's inability to simultaneously perceive high‐frequency variations encoding local discontinuities and low‐frequency information reflecting global scaffold changes. Moreover, different chemical views exhibit distinct spectral characteristics. For instance, the scaffold graph predominantly encodes global topology and concentrates its signal in the low‐frequency domain, whereas the pharmacophore graph emphasizes a few critical interaction sites, resulting in high‐frequency information corresponding to abrupt local property shifts. By integrating these complementary views and applying spectral decomposition, the PrismNet framework effectively disentangles property‐relevant features at different frequency scales from the composite molecular signal.

To enhance the model's sensitivity to both structural variations and global patterns within molecular graphs, we introduce a spectral‐domain decomposition mechanism that separates node features into high‐frequency and low‐frequency information, and employ dedicated filters to model each frequency band accordingly.

In spectral graph theory, the standard graph Laplacian matrix [78] *L* is defined as *L*  =  *D* − *A*, where *A* is the adjacency matrix of the molecular graph, and *D* is the degree matrix whose diagonal entries satisfy $D_{ii}=\sum_{j=1}^{n}A_{ij}$, representing the degree (i.e., the number of connections) of a node *v_i_*. This matrix encodes the structural relationships among nodes in the graph. To eliminate the influence of node‐degree variability, we define the symmetrically normalized graph Laplacian. $L_{norm}=D^{-\frac{1}{2}}\;L\;D^{-\frac{1}{2}}=I-D^{-\frac{1}{2}}AD^{-\frac{1}{2}}$ and denote the normalized adjacency matrix as $P=D^{-\frac{1}{2}}\;AD^{-\frac{1}{2}}$, so that *L_norm_* =  *I* − *P*. Since *L_norm_* is real and symmetric, it admits an orthogonal decomposition: *L_norm_* =  *U*Λ*U^T^*, where *U*  = [*u*_1_,⋅⋅⋅, *u_n_*]  comprises orthonormal eigenvectors and Λ  =  *diag*(λ_1_,⋅⋅⋅, λ_*n*_) contains their corresponding eigenvalues. Each $\lambda_{i}\in \left[0,2\right]$ [79], and larger λ_*i*_ indicates a basis function *u_i_* with more rapid variation over the graph—that is, a higher frequency. The graph Fourier transform projects a signal $x\in R^{n}$ onto the eigenvector basis as $\hat{x}=U^{T}\;x$, and the inverse transform is given by $x=U\hat{x}$ [80, 81].

The convolution of two time domain signals equals the element‐wise product of their Fourier transforms in the frequency domain, followed by an inverse Fourier transform: $f*g=F^{-1}\;\left(F(f)⊙F(g)\right)$, where ⊙ denotes the Hadamard (element‐wise) product. Adapting this to graphs, the spectral graph convolution is defined by: $g{*}_{G}x=U\;\left(g\left(\Lambda \right)⊙\left(U^{T}x\right)\right)=Ug\left(\Lambda \right)U^{T}x$. Here, *g* (Λ) =  *diag*(*g*(λ_1_),⋅⋅⋅, *g*(λ_*n*_)) is the spectral filter, and *x* is the input graph signal.

High‐frequency components correspond to fine‐grained structural information embedded in molecules—for instance, the localized electronic effects exerted by strongly electron‐withdrawing groups (e.g., nitro or fluorine atoms) on neighboring atoms, or the steric strain induced by unique spatial conformations such as bridged ring systems. The high‐frequency filter is designed to capture these rapidly varying, locally specific features within a molecule. This includes sharp changes in node attributes arising from distinct chemical bonds (e.g., double or triple bonds) or pronounced steric hindrance—factors often critical for determining chemical specificity and target selectivity. Additionally, it targets unique intra‐group environments, such as those found within functional groups like ‐NO_2_ or ‐SO_3_H, and key interatomic interactions that directly govern molecular properties. This high‐frequency information is indispensable for distinguishing molecules with high structural similarity but markedly different physicochemical or biological properties.

For the high‐frequency filter, we define the spectral filtering function as *g_high_* (λ) =  λ, thereby preserving high‐eigenvalue components and attenuating low‐frequency ones. The high‐frequency information is given by: $x^{high}=g_{high}\;{*}_{G}x=L_{norm}\;x=\left(I-P\right)\;x$. The *i*‐th component is:

$$x_{i}^{high}=\sum_{k=1}^{n}{\left(I-P\right)}_{ik}x_{k}=x_{i}\;-\sum_{k=1}^{n}P_{ik}x_{k},$$

$$P_{ik}={\left(D^{-\frac{1}{2}}AD^{-\frac{1}{2}}\right)}_{ik}=\sum_{u}\sum_{v}{\left(D^{-\frac{1}{2}}\right)}_{iu}\;A_{uv}{\left(D^{-\frac{1}{2}}\right)}_{vk}$$

Thus, we can derive the following expression for the normalized adjacency matrix element *P_ik_*:

$$P_{ik}=\left\{\begin{array}{ccc} \frac{1}{\sqrt{\left|N_{i}\right|\left|N_{k}\right|}}, & \mathrm{if}\;k\in N_{i}, & \\ 0, & else & \end{array}\right.$$

where $N_{i}$ denotes the set of neighbors of node *i*, and $|N_{i}|$ represents the degree of node *i*. Therefore, the high‐frequency component at node *i* can be written as:

(4) $$x_{i}^{\mathrm{high}}=x_{i}\;-\sum_{j\in N_{i}}\frac{x_{j}}{\sqrt{\left|N_{i}\right|\left|N_{j}\right|}}$$

In contrast, low‐frequency components correspond to the “smooth” or “global” structural patterns within a molecule. The low‐frequency filter focuses on broad, gradually varying topological features, such as extended carbon chains or conjugated systems like aromatic rings. By aggregating information from neighboring atoms, it captures the collective properties of molecular subregions and effectively captures the overall configurational coherence, scaffold continuity, and large‐scale physicochemical characteristics of the molecule. Accordingly, we employ low‐frequency filters to enhance the model's capacity for capturing global structural trends at the molecular scale.

For the low‐frequency filter, we define the spectral filtering function as *g_low_* (λ) =  2 − λ. When λ is small (corresponding to low‐frequency information), 2 − λ is large, thus amplifying smooth components; conversely, when λ is large, 2 − λ becomes small, suppressing high‐frequency variations. $x^{low}=g_{low}\;{*}_{G}x=\left(2I-L_{norm}\right)\;x=\left(I+P\right)\;x$, the *i*‐th component is given by:

$$x_{i}^{low}=\sum_{k=1}^{n}{\left(I+P\right)}_{ik}x_{k}=x_{i}+\sum_{k=1}^{n}P_{ik}x_{k}$$

Similarly, we obtain the following expression for the low‐frequency information at node *i*:

(5) $$x_{i}^{low}=x_{i}+\sum_{j\in N_{i}}\frac{x_{j}}{\sqrt{\left|N_{i}\right|\left|N_{j}\right|}}$$

The spectral enhancement module is centered around an attention mechanism, which is integrated with frequency‐specific information extraction to yield more expressive node representations. Specifically, given the input node feature matrix *X*, we first compute the query, key, and value matrices:

(6) $$\begin{array}{c} Q=W^{Q}\;h\left(X\right)+HighFreq\left(h\left(X\right)\right),\\ K=W^{K}\;h\left(X\right)+LowFreq\left(h\left(X\right)\right),\\ V=W^{V}\;h\left(X\right) \end{array}$$

Here, *W^Q^*, *W^K^*, and *W^V^* are learnable parameter matrices. The attention score matrix is computed as:

(7) $$S=softmax\left(\frac{QK^{T}}{\sqrt{d_{k}}}\right)$$

where $d_{k}=\frac{d}{h}$ is the dimension of each attention head. To capture information diffusion over larger neighborhoods, we introduce the higher‐order Laplacian matrix *L^k^*, which is the *k*‐th power of the standard Laplacian *L* and reflects global structural relationships within the *k*‐hop neighborhood. To incorporate this global structure and dynamic message‐attenuation mechanism, we adjust the attention matrix using. *L^k^* to construct an enhanced message matrix:

(8) $$M=\frac{S+\alpha L^{k}⊙A}{J+\beta \cdot A}\;$$

Here, *J* is a matrix of all ones with the same dimensions as *A*; α and β are tunable parameters, and ⊙ denotes element‐wise multiplication. This operation is an element‐wise division. The attention score matrix *S* captures feature‐based relevance, while the higher‐order Laplacian term modulates the attention based on topological distances between nodes. We introduce a dynamic message‐attenuation mechanism that scales down propagation strength with increasing node distance, thereby mitigating GNN oversmoothing. This mechanism enables selective focus on both local and long‐range interactions, emulating the chemical principle that short‐range effects dominate while distant influences fade. Consequently, the model emphasizes nearby atoms yet retains attenuated global context.

Using the resulting message matrix *M*, we update the node features according to the following rule:

(9) $$H^{\prime }=MV+HighFreq\left(h\left(X\right)\right)+LowFreq\left(h\left(X\right)\right)$$

### 4.3 Dynamic Task Weighting

Let $L_{i}$ denote the individual loss of task *i*, and let λ_*i*_ be its corresponding weight. We dynamically update the task weights based on the deviation between each task's current loss and its historical average loss. The update rule is given by:

(10) $$\lambda_{i}^{t+1}=\lambda_{i}^{t}\;\left(1+\gamma \frac{L_{i}^{t}-{\bar{L}}_{i}}{{\bar{L}}_{i}+\varepsilon }\right)$$

where $\lambda_{i}^{t}$ is the weight of task *i* at iteration *t*, $L_{i}^{t}$ is its current loss, ${\bar{L}}_{i}$ is the historical average loss, γ is a hyperparameter controlling the adjustment magnitude, and ε is a small constant to prevent division by zero. The normalized task weight of task *i* is defined as follows:

(11) $${\tilde{\lambda }}_{i}=\frac{\lambda_{i}}{\sum_{j=1}^{T}\lambda_{j}}\;$$

where *T* is the total number of tasks. The effectiveness and stability of this weighting scheme are visualized in Figure S5, which shows the task weight evolution on the ClinTox dataset.

Let *N_c_* denote the number of samples in class *c* within the training set. The class weight is computed as:

(12) $$w_{c}=\frac{1-\beta }{1-\beta^{N_{c}}}\;$$

where β∈(0,1) is a hyperparameter used to regularize the class weights. When β is close to 1, the dynamic range of the weights becomes smaller, making it suitable for scenarios with highly imbalanced class distributions. To maintain a comparable scale of loss across different class distributions and ensure numerical stability during training, we normalize the class weights such that their sum equals the total number of classes *C*, following the recommendation of Cui et al [82]. The normalization is defined as follows:

(13) $$\tilde{w_{c}}=\frac{w_{c}}{\sum_{c^{\prime }=1}^{C}w_{c^{\prime }}}\;\cdot C$$

In the presence of class imbalance, we apply a class‐weighted correction to the loss using the computed class weights $\tilde{w_{c}}$. Specifically, the weight for positive samples is $\tilde{w_{1}}$ and for negative samples is $\tilde{w_{0}}$. The adjusted loss function for each sample is defined as:

(14) $$L_{i}=-\tilde{w_{1}}\cdot y_{i}\log\left(p_{i}\right)-\tilde{w_{0}}\cdot \left(1-y_{i}\right)\log\left(1-p_{i}\right)$$

The total classification loss is then given by:

(15) $$L_{\mathrm{classification}}=\sum_{i=1}^{T}{\tilde{\lambda }}_{i}L_{i}\;$$

We introduce a contrastive learning loss. Let the feature representation of sample *i* be denoted as $h_{i}\in R^{d}$. The similarity between sample *i* and sample *j* is defined as:

$$sim\;\left(h_{i},h_{j}\right)=\frac{h_{i}^{T}h_{j}}{∥h_{i}∥∥h_{j}∥}$$

The similarity matrix $S\in R^{N\times N}$ captures pairwise similarity between all samples, where *N* is the total number of samples. The contrastive learning loss is defined as:

(16) $$L_{contrastive}=-\frac{1}{N}\sum_{i=1}^{N}\log\frac{\sum_{j\in P\left(i\right)}\exp\left(sim\left(h_{i},h_{j}\right)/\tau \right)}{\sum_{k=1}^{N}\exp\left(sim\left(h_{i},h_{k}\right)/\tau \right)}$$

where P(i) denotes the set of positive samples for sample *i*, and τ is a temperature parameter that controls the sharpness of the similarity distribution. The joint optimization of dynamic task weighting and contrastive learning loss enables the model to excel in handling multi‐task learning and class imbalance challenges, thereby achieving accurate molecular property prediction.

Finally, we combine the contrastive learning loss with the classification loss to form the total loss function:

(17) $$L_{total}=L_{classification}+\lambda \cdot L_{contrastive}$$

where λ is a weighting hyperparameter that controls the contribution of the contrastive loss in the overall optimization objective.

For the readout module, we design a GRU structure equipped with a dynamic message aggregation mechanism. The bidirectional GRU captures richer contextual information among nodes, and its output is combined with a linearly projected and Mish‐activated representation through residual connections and normalization. The final output is then used for downstream task prediction. Training procedures and hyperparameter configurations are provided in the “Training details and hyperparameters” section of the Supplementary Information.

### 4.4 Statistical Analysis

All experimental results were derived from three independent model runs to ensure the reliability of the findings. In this work, all reported performance metrics are presented as “mean ± standard deviation”, where the standard deviation quantifies the dispersion of results across these three independent runs. Error bars in all figures consistently represent the standard deviation across these three independent runs. For regression tasks, we employed the root mean square error (RMSE) or mean absolute error (MAE) as the evaluation metric, while for classification tasks, we used the area under the receiver operating characteristic curve (ROC‐AUC) or the area under the precision‐recall curve (AUPRC) to assess model performance.

## Author Contributions

Y.L. conceived and designed the project. C.X. designed and performed the experiments. C.X. analyzed the results data and drew figures. Y.L. conceived the writing main framework. C.X. finished the original draft with elegant figures. Y.L. and Q.W. provided critical feedback for shaping the research and manuscript. Q.W. managed and supervised the project. All authors discussed the results and contributed to the final manuscript.

## Funding

This research was supported by the National Natural Science Foundation of China (No. 32560689), Natural Science Special Fund of Guizhou University (No. 202409), National Key R&D Program of China (2024YFD2001100, 2024YFE0214300), the National Natural Science Foundation of China (No. 62162008), Guizhou Provincial Science and Technology Projects ([2024]002, CXTD[2023]027), Guizhou Province Youth Science and Technology Talent Project ([2024]317), Guiyang Guian Science and Technology Talent Training Project ([2024] 2–15).

## Conflicts of Interest

The authors declare no conflicts of interest.

## Supporting information




**Supporting File**: advs73448‐sup‐0001‐SuppMat.docx.

## Data Availability

All experimental datasets employed in this research are sourced from openly available repositories, namely MoleculeNet [36] (https://moleculenet.org/datasets‐1), Therapeutics Data Commons [37] (https://tdcommons.ai/benchmark/overview), FS‐Mol [38] (https://github.com/microsoft/FS‐Mol), and activity cliff datasets [29, 71] (https://doi.org/10.6084/m9.figshare.28748252.v1). The code is available on Github: https://github.com/GZU-SAMLab/PrismNet.
