# Supplementary material for: Spectral Decomposition of Chemical Semantics for Activity Cliffs‐Aware Molecular Property Prediction
Source: Adv Sci (Weinh). 2026 Feb 3;13(13):e17579. doi: 10.1002/advs.202517579 (PMC12955929; doi:10.1002/advs.202517579)
Supplement: Supplementary file 1 — Supporting File: advs73448‐sup‐0001‐SuppMat.docx. [file ADVS-13-e17579-s001.docx]

Supporting Information

Spectral Decomposition of Chemical Semantics for Activity Cliffs-Aware Molecular Property Prediction

Chaoyang Xie, Junhu Xu, Guangyi Huang, Shihang Wang, Mutian He, Xinyu Dong, Huiyang Hong, Xiaojun Yao, Qi Wang*, and Yuquan Li*

**Details of Datasets**

**Supplementary Table 1.** Overview of the key characteristics and statistical properties of the MoleculeNet benchmark datasets used for model evaluation.

| **Dataset** | **Number of tasks** | **Task type** | **Number of molecules** | **Sample split (train/valid/test)** | **Describe** |
| --- | --- | --- | --- | --- | --- |
| BACE^[13]^ | 1 | Classification | 1513 | 1211/151/151 | Binding results of human BACE-1 inhibitors |
| BBBP^[14]^ | 1 | Classification | 2039 | 1631/204/204 | Blood-brain barrier penetration |
| HIV^[1]^ | 1 | Classification | 41127 | 32901/4113/4113 | The ability to suppress HIV replication |
| ClinTox^[15,16]^ | 2 | Classification | 1478 | 1182/148/148 | Clinical trial toxicity and FDA approval status |
| MUV^[17]^ | 17 | Classification | 93087 | 74469/9309/9309 | A subset of PubChem BioAssay |
| SIDER^[18,19]^ | 27 | Classification | 1427 | 1141/143/143 | Adverse drug reactions to the 27 systemic organs |
| Tox21^[20]^ | 12 | Classification | 7831 | 6265/783/783 | Qualitative toxicity measurements |
| ToxCast^[21]^ | 617 | Classification | 8575 | 6859/858/858 | Toxicology data based on in vitro screening |
| ESOL^[22]^ | 1 | Regression | 1128 | 902/113/113 | Water solubility |
| FreeSolv^[23]^ | 1 | Regression | 642 | 513/64/64 | Hydrogen free energy |
| Lipophilicity^[24]^ | 1 | Regression | 4200 | 3360/420/420 | Octanol/water distribution ratio, coefficient |

MoleculeNet^[1]^ is a widely used benchmark platform for molecular property prediction and drug discovery research, encompassing a diverse collection of datasets across multiple domains. Among them, eleven datasets including BACE serve as key resources for drug discovery, covering various aspects such as target bioactivity, drug delivery characteristics, and toxicity evaluation. These datasets offer comprehensive data support spanning from early-stage drug discovery to safety assessment. Their diversity and richness enable researchers to investigate molecular properties from multiple perspectives, optimize drug design, and ultimately enhance the success rate of pharmaceutical development.

The FS-Mol^[2]^ dataset, released by Microsoft, is a benchmark for few-shot molecular learning. We randomly selected nine bioactivity classification tasks from this dataset, each containing approximately 80–200 molecular samples. These subsets are used to evaluate the model's generalization ability in data-scarce scenarios, targeting the prediction of activity against various drug targets such as TANK1, DHFR, and JAK2. Additionally, we conducted benchmark evaluations on a range of ADMET datasets provided by the Therapeutics Data Commons (TDC).^[3]^

To ensure fair comparison, we adopt a scaffold-based data splitting strategy, dividing each dataset into training, validation, and test sets with an 8:1:1 ratio.^[4-6]^ This scaffold-based approach effectively simulates the influence of molecular structural variations on property prediction and facilitates the evaluation of model performance under diverse structural distributions. For classification tasks, we use the area under the receiver operating characteristic curve (ROC-AUC) as the evaluation metric, as it captures the trade-off between true positive rate and false positive rate across varying thresholds. ROC-AUC provides a comprehensive assessment of classification performance, particularly under class imbalance. For regression tasks, we use root mean square error (RMSE) to quantify the average deviation between the predicted values and ground truth.





**Supplementary Figure 1.** Severe class imbalance is common across benchmark datasets used in drug discovery. We provide an intuitive visualization of the class distribution across multiple tasks in five widely used molecular property prediction benchmarks. **a** The SIDER dataset displays the number of samples in each class across 27 tasks using stacked bar plots, labeled as Task 1-Task 27. **b** The Tox21 dataset uses grouped bar plots to highlight the pronounced class imbalance in various tasks. **c** The MUV dataset presents the class imbalance ratio (Class 0 / Class 1) for each task using bar plots. **d** The ClinTox dataset visualizes the proportion of the two classes in its two tasks using pie charts. **e** The ToxCast dataset employs a box plot to show the distribution of class imbalance ratios across 617 tasks, revealing a large number of highly imbalanced tasks.

**Notations and Preliminary**

In molecular property prediction tasks, we model each molecule as an undirected graph $\mathcal{G=}\left( \mathcal{V, E} \right)$ , where $\mathcal{V}$ is the set of nodes representing atoms in the molecule (i.e., $\left| \mathcal{V} \right|=n$). $\mathcal{E}$ is the set of edges representing chemical bonds between atoms, with $\left| \mathcal{E} \right|=m$ indicating the number of edges. $X\in\mathbb{R}^{n\times d_{x}}$ is the node feature matrix, where $d_{x}$ denotes the dimensionality of each node's feature vectors. For each node $v_{i}$, the corresponding row $x_{i}$ in $X$ is a $d_{x}$ -dimensional real-valued vector encoding the atom's chemical properties (e.g., atom type and partial charge). $A\in\mathbb{R}^{n\times n}$ is the adjacency matrix of the molecular graph.

To capture global molecular information, we introduce a special global information node whose features are computed from molecular fingerprints and descriptors,^[7,8]^ denoted by $h_{k}\in\mathbb{R}^{d_{atom}}$. The global information node is then connected to every atom node, yielding an extended graph $\mathcal{G}_{k}$ whose adjacency matrix is:

$$A_{k}=\left[ \begin{matrix} A & 1 \\ 1^{T} & 0 \end{matrix} \right]$$

where $1\in\mathbb{R}^{n\times1}$ is a column vector of ones denoting its links to all atom nodes. This additional node thus enables effective capture of a molecule's global structural and physicochemical characteristics. Next, we define the degree matrix $D\in\mathbb{R}^{\left( n+1 \right)\times\left( n+1 \right)}$ for the extended graph, whose diagonal entries satisfy $D_{ii}=\sum_{j=1}^{n} A_{ij}$ , representing the degree (i.e., the number of connections) of node $v_{i}$.

**Supplementary Table 2.** Detailed definitions of atomic and chemical bond features used for initializing molecular graph representations. This table enumerates and describes all atom-level and bond-level features employed in this study to convert molecular structures into graph neural network inputs. These carefully crafted features collectively form the initial node and edge feature matrices, providing a rich informational foundation for the model to learn complex structure-activity relationships from the underlying chemical structures.

| **Type** | **Feature** | **Description** |
| --- | --- | --- |
| Atom Features | Atomic Number | One-hot encoding of the atomic number ranging from 1 to 100 (including unknown), which uniquely identifies the type of atom. |
|  | Hybridization | One-hot encoding of the hybridization type of the atom (SP, SP2, SP3, SP3D, SP3D2).(including unknown) |
|  | Total Number of Hydrogens | Total number of hydrogen atoms attached to the atom, normalized by dividing by 8.(float) |
|  | Degree | Number of bonds connected to the atom, normalized by dividing by 4.(float) |
|  | Formal Charge | Formal charge of the atom, normalized by dividing by 8.(float) |
|  | Total Valence | Total valence electrons of the atom, normalized by dividing by 8.(float) |
|  | Gasteiger Charge | Gasteiger partial charge of the atom, with NaN or inf values replaced by 0.(float) |
|  | Gasteiger HCharge | Gasteiger partial charge of the hydrogen atoms attached to the atom, with NaN or inf values replaced by 0.(float) |
|  | Is Aromatic | Whether the atom is part of an aromatic system (1 if true, 0 otherwise).(binary) |
|  | Is In Ring | Whether the atom is part of a ring structure (1 if true, 0 otherwise).(binary) |
|  | Index for Position Encoding | Index of the atom in the molecule, starting from 1.(integer) |
| Bond Features | Bond Type | One-hot encoding of the bond type (SINGLE, DOUBLE, TRIPLE, AROMATIC). |
|  | Stereochemistry | One-hot encoding of the bond stereochemistry (STEREOANY, STEREOCIS, STEREOE, STEREONONE, STEREOTRANS, STEREOZ). |
|  | Is Conjugated | Whether the bond is conjugated (1 if true, 0 otherwise).(binary) |
|  | Is Aromatic | Whether the bond is part of an aromatic system (1 if true, 0 otherwise).(binary) |
|  | Is In Ring | Whether the bond is part of a ring structure (1 if true, 0 otherwise).(binary) |

**Training Details and Hyperparameters**

To ensure the reliability of the results, we performed three independent runs with different random seeds. We evaluated classification performance using the area under the ROC curve (ROC-AUC) and regression performance using root mean square error (RMSE). All experiments were implemented in PyTorch 1.10.0^[9]^ with Python 3.7.13 and executed on a Linux server equipped with an NVIDIA A40 GPU.

Our graph neural network employs a five-layer encoder, each layer featuring four self-attention heads with a head dimension of 64 (yielding a hidden size of 256). This architecture balances fine-grained local detail capture with computational efficiency. The feed-forward neural network comprises a single layer with the Mish activation function for nonlinearity. To mitigate overfitting, we apply dropout with a rate of 0.2 throughout the model.

During data preprocessing, we employed RDKit^[10]^ to convert raw molecular data into graph structures. Each atom is represented by a 115-dimensional feature vector encoding physicochemical and topological properties, and each bond by a 13-dimensional feature vector.

For model training, we employed the Adam optimizer^[11]^ and implemented a learning rate scheduling strategy based on a warmup scheme. To quickly stabilize model parameters, the warmup phase was set to span 30 epochs, with the total number of warmup steps computed based on the training set size. The warmup factor was set to 0.2, and a custom scheduler was used to dynamically adjust the learning rate. This strategy allows the model to learn stably with a small learning rate during the early stages, followed by an inverse square root decay as training progresses—effectively mitigating parameter instability in the early phase and reducing the risk of overfitting in later stages.

To balance computational efficiency and memory consumption, the batch size was set to 32. Early stopping was triggered if validation performance did not improve for 50 consecutive epochs. The maximum number of training epochs was set to 300. For efficient data loading, we utilized eight subprocesses to enable parallel data processing.

During the pre-training phase, to enable the model to learn general-purpose molecular representations from a large-scale unlabeled chemical space, we randomly sampled 250,000 unlabeled molecules from the ZINC15^[12]^ dataset as the pre-training corpus. To ensure strict data independence and prevent potential information leakage, we conducted a systematic check between this pre-training dataset and all 64 downstream benchmark datasets. Exact Matching revealed only 20 overlapping molecules out of 419,304 benchmark samples (0.0048%), with 59 out of 64 datasets showing zero overlap. Furthermore, a Tanimoto similarity analysis (Mean Max-Similarity ≈ 0.292) confirmed that the downstream tasks occupy a chemical space distinct from the pre-training set. These findings, combined with our scaffold-based splitting strategy, ensure that the model's performance reflects genuine transfer learning rather than memorization. The pre-training process was conducted on a single NVIDIA A40 GPU and converged after approximately five days. The resulting pre-trained model parameters were then fine-tuned on downstream tasks.

**Multi-View Generation Algorithm**

| **Algorithm:** Generation of Chemical Semantic Views in PrismNet | |
| --- | --- |
|  | **Input:** Molecular graph $\mathcal{G}=\left( \mathcal{V},\mathcal{E} \right)$ with $L=\left\vert\mathcal{V} \right\vert$ atoms **Output:** Three complementary semantic views: $\mathcal{G}_{s},\mathcal{G}_{f},\mathcal{G}_{p}$ |
|  |  |
| 1 | **Step 1: Graph initialization** |
| 2 | Compute atom features $X\in\mathbb{R}^{L\times d_{atom}}$ using atomic descriptors: **atom.GetAtomicNum(), atom.GetHybridization(), atom.GetTotalNumHs(), atom.GetFormalCharge()**, etc.; |
| 3 | Compute bond features $B\in\mathbb{R}^{L\times L\times d_{edge}}$ using bond-level properties: **bond.GetBondType(), bond.GetIsConjugated(), bond.GetStereo()**, etc.; |
| 4 | Compute adjacency matrix $A\in\mathbb{R}^{L\times L}$ via **rdkit.Chem.rdmolops.GetAdjacencyMatrix(**$\mathcal{G}$**)**; |
| 5 | Generate global descriptor $X_{g}$ using: |
| 6 | **smiles = rdkit.Chem.MolToSmiles(**$\mathcal{G}$**)** |
| 7 | **mol2d = RDKit2DNormalized(Chem.MolFromSmiles(smiles));** |
| 8 | Append global information node $X_{g}$ to $X$ as $\left( L+1 \right)$ -th row; |
| 9 | update $A$ and $B$ by connecting it to all existing nodes; |
|  |  |
| 10 | **Step 2: Scaffold graph generation** |
| 11 | $M_{\text{s}}$ $\leftarrow$ **rdkit.Chem.Scaffolds.MurckoScaffold.GetScaffoldForMol(**$\mathcal{G}$**)**; |
| 12 | $I_{\text{s}}$ $\leftarrow\mathcal{G}$**.GetSubstructMatch(**$M_{\text{s}}$**)**; |
| 13 | Construct binary mask $m\in\{0,1{\}}^{L+1}$ where $m_{i}=1$ if $i\in I_{\text{s}}$ , else $0$; |
| 14 | Force $m_{L+1}=1$ to retain global information node; |
| 15 | $X^{s}=X\odot m$, $A^{s}=A\odot\left( mm^{\top} \right)$, $B^{s}=B\odot\left( mm^{\top} \right)$; |
|  |  |
| 16 | **Step 3: Functional group graph generation** |
| 17 | Load SMARTS patterns $\{S_{1},\cdots,S_{K}\}$ from predefined library; |
| 18 | Initialize $I_{f}=\emptyset$; |
| 19 | **foreach** $S_{i}$ in SMARTS library **do** |
| 20 | $P_{i}$ $\leftarrow$**rdkit.Chem.MolFromSmarts(**$S_{i}$**)**; |
| 21 | $M_{i}$ $\leftarrow\mathcal{G}$**.GetSubstructMatches(**$P_{i}$**)**; |
| 22 | $I_{f}\leftarrow I_{f}\cup M_{i}$; |
| 23 | Construct mask $m\in\{0,1{\}}^{L+1}$: $m_{i}=1$ if $i\in I_{f}$ , else $0$; set $m_{L+1}=1$; |
| 24 | $X^{f}=X\odot m$, $A^{f}=A\odot\left( mm^{\top} \right)$, $B^{f}=B\odot\left( mm^{\top} \right)$; |
|  |  |
| 25 | **Step 4: Pharmacophore graph generation** |
| 26 | $F\leftarrow$ **rdkit.Chem.ChemicalFeatures.BuildFeatureFactory("BaseFeatures.fdef")**; |
| 27 | $\mathcal{F}_{\mathcal{G}}\leftarrow F$**.GetFeaturesForMol(**$\mathcal{G}$**)**; |
| 28 | $I_{p}\leftarrow\bigcup_{f\in\mathcal{F}_{\mathcal{G}}} f.GetAtomIds\left( \right)$; |
| 29 | Construct mask $m\in\{0,1{\}}^{L+1}$: $m_{i}=1$ if $i\in I_{p}$, else $0$; set $m_{L+1}=1$; |
| 30 | $X^{p}=X\odot m$, $A^{p}=A\odot\left( mm^{\top} \right)$, $B^{p}=B\odot\left( mm^{\top} \right)$; |
|  |  |
| 31 | **return** $\mathcal{G}_{s}= \left( X^{s},A^{s},B^{s} \right)$, |
| 32 | $\mathcal{G}_{f}=\left( X^{f},A^{f},B^{f} \right)$, |
| 33 | $\mathcal{G}_{p}=\left( X^{p},A^{p},B^{p} \right)$ |

**Comparison with Baselines**

**Supplementary Table 3.** PrismNet exhibits highly competitive performance across multiple TDC molecular absorption property prediction tasks. The table includes both classification tasks (evaluated by ROC-AUC) and regression tasks (evaluated by MAE), with all results reported in the format of “mean ± standard deviation”. Arrows (↑ or ↓) next to each task name indicate whether a higher or lower value is preferred. For each dataset, the best result is highlighted in bold, and the second-best is underlined. A dash “-” indicates that the corresponding result was not reported in the original publication.

| Datasets | Caco2_Wang↓ | Pgp_Broccatelli↑ | Bioavailability_Ma↑ | Lipophilicity_AstraZeneca↓ | Solubility_AqSolDB↓ |
| --- | --- | --- | --- | --- | --- |
| Metrics | MAE | ROC-AUC | ROC-AUC | MAE | MAE |
| Morgan + MLP (DeepPurpose)^[25]^ | 0.908 ± 0.060 | 0.880 ± 0.006 | 0.581 ± 0.086 | 0.701 ± 0.009 | 1.203 ± 0.019 |
| GCN^[26]^ | 0.599 ± 0.104 | 0.895 ± 0.021 | 0.566 ± 0.115 | 0.541 ± 0.011 | 0.907 ± 0.020 |
| AttrMasking^[27]^ | 0.546 ± 0.052 | - | 0.577 ± 0.087 | 0.547 ± 0.024 | 1.026 ± 0.020 |
| NeuralFP^[28]^ | 0.530 ± 0.102 | 0.902 ± 0.020 | 0.632 ± 0.036 | 0.563 ± 0.023 | 0.947 ± 0.016 |
| ContextPred^[27]^ | 0.502 ± 0.036 | - | 0.671 ± 0.026 | 0.535 ± 0.012 | 1.040 ± 0.045 |
| CNN (DeepPurpose)^[25]^ | 0.446 ± 0.036 | 0.908 ± 0.012 | 0.613 ± 0.013 | 0.743 ± 0.020 | 1.023 ± 0.023 |
| AttentiveFP^[29]^ | 0.401 ± 0.032 | 0.892 ± 0.012 | 0.632 ± 0.039 | 0.572 ± 0.007 | 0.776 ± 0.008 |
| RDKit2D + MLP(DeepPurpose)^[25]^ | 0.393 ± 0.024 | 0.918 ± 0.007 | 0.672 ± 0.021 | 0.574 ± 0.017 | 0.827 ± 0.047 |
| Chemprop^[30]^ | 0.340 ± 0.016 | 0.858 ± 0.036 | 0.579 ± 0.023 | - | 0.818 ± 0.010 |
| Chemprop-RDKit^[30]^ | 0.328 ± 0.024 | 0.883 ± 0.016 | 0.668 ± 0.069 | - | 0.762 ± 0.020 |
| Basic ML^[31]^ | 0.321 ± 0.005 | 0.818 ± 0.000 | 0.523 ± 0.011 | 0.617 ± 0.003 | 0.828 ± 0.002 |
| PrismNet | **0.301 ± 0.014** | **0.920 ± 0.014** | **0.757 ± 0.020** | **0.519 ± 0.005** | **0.681 ± 0.015** |

**Supplementary Table 4.** PrismNet demonstrates highly competitive performance on tasks related to molecular distribution prediction. The table includes both classification tasks (evaluated using ROC-AUC) and regression tasks (evaluated using MAE), with all results reported in the format of “mean ± standard deviation”. Arrows (↑ or ↓) next to each task name indicate whether a higher or lower value is preferable. For each dataset, the best result is highlighted in bold, and the second-best is underlined. A dash “-” denotes that the corresponding result was not reported in the original publication.

| Datasets | BBB_Martins↑ | PPBR_AZ↓ |
| --- | --- | --- |
| Metrics | ROC-AUC | MAE |
| RDKit2D + MLP (DeepPurpose)^[25]^ | 0.889 ± 0.016 | 9.994 ± 0.319 |
| NeuralFP^[28]^ | 0.836 ± 0.009 | 9.292 ± 0.384 |
| Morgan + MLP (DeepPurpose)^[25]^ | 0.823 ± 0.015 | 12.848 ± 0.362 |
| GCN^[26]^ | 0.842 ± 0.016 | 10.194 ± 0.373 |
| ContextPred^[27]^ | **0.897 ± 0.004** | 9.445 ± 0.224 |
| CNN (DeepPurpose)^[25]^ | 0.781 ± 0.030 | 11.106 ± 0.358 |
| Chemprop-RDKit^[30]^ | 0.868 ± 0.027 | 8.312 ± 0.180 |
| Chemprop^[30]^ | 0.820 ± 0.112 | 7.811 ± 0.163 |
| Basic ML^[31]^ | 0.811 ± 0.013 | 9.185 ± 0.000 |
| AttrMasking^[27]^ | 0.892 ± 0.012 | 10.075 ± 0.202 |
| AttentiveFP^[29]^ | 0.855 ± 0.011 | 9.373 ± 0.335 |
| BaseBoosting K^[32]^ | - | 7.914 ± 0.096 |
| PrismNet | 0.894 ± 0.008 | **7.600 ± 0.323** |

**Supplementary Table 5.** PrismNet demonstrates highly competitive performance on molecular metabolism prediction tasks. All tasks are evaluated using AUPRC (area under the precision-recall curve), and all results are reported in the format of “mean ± standard deviation”. An arrow (↑) next to each task name indicates that higher values are better. For each dataset, the best result is highlighted in bold, and the second-best is underlined. A dash “-” denotes that the corresponding result was not reported in the original publication.

| Datasets | CYP2C9_Veith↑ | CYP2D6_Veith↑ | CYP2C9_Substrate↑ | CYP2D6_Substrate↑ |
| --- | --- | --- | --- | --- |
| Metrics | AUPRC | AUPRC | AUPRC | AUPRC |
| ZairaChem^[33]^ | - | 0.644 ± 0.085 | 0.441 ± 0.033 | - |
| RDKit2D + MLP (DeepPurpose)^[25]^ | 0.742 ± 0.006 | 0.616 ± 0.007 | 0.360 ± 0.040 | - |
| NeuralFP^[28]^ | 0.739 ± 0.010 | 0.627 ± 0.009 | 0.359 ± 0.059 | 0.572 ± 0.062 |
| Morgan + MLP (DeepPurpose)^[25]^ | 0.715 ± 0.004 | 0.587 ± 0.011 | 0.380 ± 0.015 | - |
| GCN^[26]^ | 0.735 ± 0.004 | 0.616 ± 0.020 | 0.344 ± 0.051 | 0.617 ± 0.039 |
| CNN (DeepPurpose)^[25]^ | 0.713 ± 0.006 | 0.544 ± 0.053 | 0.367 ± 0.059 | 0.485 ± 0.037 |
| Chemprop^[30]^ | - | 0.645 ± 0.018 | 0.387 ± 0.012 | 0.624 ± 0.046 |
| Basic ML^[31]^ | 0.556 ± 0.000 | 0.358 ± 0.000 | 0.281 ± 0.000 | 0.478 ± 0.018 |
| AttentiveFP^[29]^ | **0.749 ± 0.004** | 0.646 ± 0.014 | 0.375 ± 0.032 | 0.574 ± 0.030 |
| PrismNet | 0.742 ± 0.007 | **0.653 ± 0.007** | **0.462 ± 0.025** | **0.664 ± 0.022** |


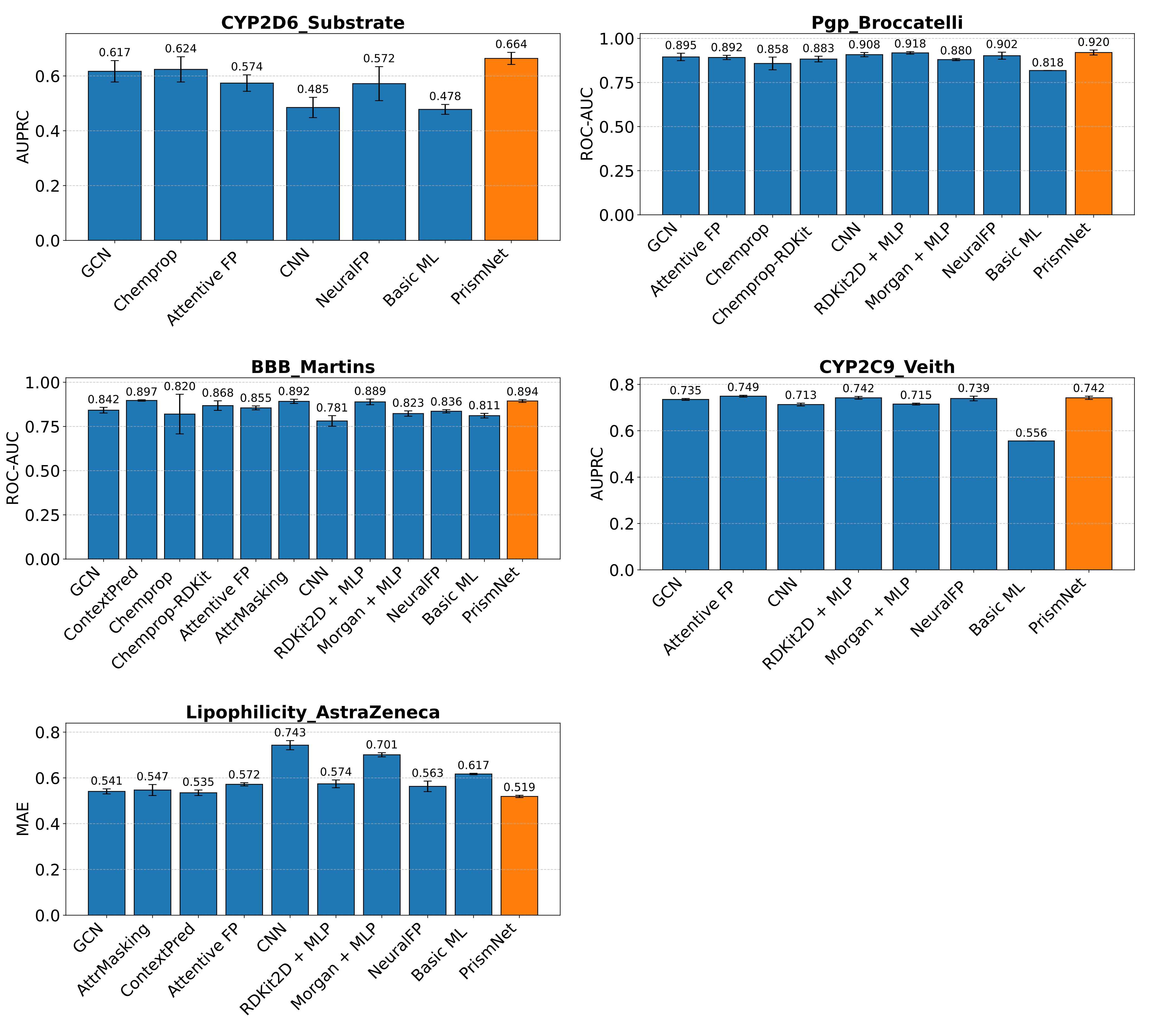


**Supplementary Figure 2.** PrismNet also demonstrates strong and competitive predictive performance on the supplementary TDC benchmark tasks. This figure, serving as a complement to the main performance results presented in Figure 2, provides an intuitive comparison between PrismNet (highlighted with a red background) and various baseline models across five additional TDC tasks. In each subplot, the vertical axis represents the evaluation metric for the task (AUPRC, ROC-AUC, or MAE), while the horizontal axis lists the competing models. The height of each bar indicates model performance—higher is better for AUPRC and ROC-AUC, and lower is better for MAE. Error bars indicate standard deviation across three independent runs.

**Supplementary Table 6.** PrismNet achieves lower overall prediction errors than mainstream baseline models across multiple quantum chemistry benchmark tasks, demonstrating strong capability in modeling molecular physical properties. This table presents a detailed comparison of PrismNet against various baseline models on three widely used quantum chemistry benchmark datasets. All tasks are evaluated using mean absolute error (MAE), where lower values indicate closer alignment with the ground truth derived from quantum chemical calculations.

| Datasets | QM7↓ | | QM8↓ | | QM9↓ | | |
| --- | --- | --- | --- | --- | --- | --- | --- |
| Molecules | 6830 | | 21786 | | 133885 | | |
| Metrics | MAE (lower is better) | | | | |  |  |
| Attentive FP^[29]^ | 72.0 | 0.0179 | | 0.00812 | | |  |
| N-Gram_RF_^[34]^ | 92.8 | 0.0236 | | 0.01037 | | |  |
| N-Gram_XBG_^[34]^ | 81.9 | 0.0215 | | 0.00964 | | |  |
| PretrainGNN^[27]^ | 113.2 | 0.0200 | | 0.00922 | | |  |
| GROVER_base_^[35]^ | 94.5 | 0.0218 | | 0.00984 | | |  |
| GROVER_large_^[35]^ | 92.0 | 0.0224 | | 0.00986 | | |  |
| MolCLR^[36]^ | 66.8 | 0.0178 | | - | | |  |
| ESM-AA^[37]^ | 60.9 | 0.0171 | | 0.00590 | | |  |
| Uni-Mol^[4]^ | 58.9 | 0.0160 | | 0.00540 | | |  |
| MOL-AE^[38]^ | 53.8 | 0.0161 | | 0.00530 | | |  |
| GCN^[26]^ | 122.9 | 0.0366 | | 0.00835 | | |  |
| GIN^[39]^ | 124.8 | 0.0371 | | 0.00824 | | |  |
| MPNN^[40]^ | 111.4 | 0.0148 | | 0.00522 | | |  |
| DMPNN^[30]^ | 103.5 | 0.0156 | | 0.00514 | | |  |
| Hu et.al^[27]^ | 113.2 | 0.0215 | | 0.00922 | | |  |
| GEM^[41]^ | 60.0 | 0.0163 | | 0.00562 | | |  |
| PrismNet | **53.0** | **0.0114** | | **0.00511** | | |  |

**Ablation Studies**

**Supplementary Table 7.** The detailed numerical results of the ablation studies confirm that each core component of PrismNet is essential for achieving optimal performance across various tasks. This table presents the complete set of performance metrics from the ablation study conducted on the MoleculeNet benchmark, quantifying the contribution of each key module in the model. Arrows (↑ or ↓) next to each dataset name indicate whether a higher or lower value is preferred. All reported results are in the format of “mean ± standard deviation”. The ablation variants are defined as follows: removing high-frequency information (w/o HF), removing low-frequency information (w/o LF), removing both high- and low-frequency information (w/o HF&LF), removing class imbalance handling (w/o CIH), removing dynamic task weighting (w/o DTW), removing message attenuation (w/o MA), removing contrastive learning (w/o CL), and removing pre-training (w/o PT).

| Datasets | BACE↑ | BBBP↑ | ClinTox↑ | HIV↑ | MUV↑ | SIDER↑ | Tox21↑ | ToxCast↑ | ESOL↓ | FreeSolv↓ | Lipophilicity↓ |
| --- | --- | --- | --- | --- | --- | --- | --- | --- | --- | --- | --- |
| Metrics | ROC-AUC (higher is better) | | | | | | | | RMSE (lower is better) | | |
| w/o HF | 0.948 ± 0.001 | 0.980 ± 0.005 | **0.997 ± 0.001** | 0.829 ± 0.011 | 0.790 ± 0.027 | 0.662 ± 0.006 | 0.845 ± 0.016 | 0.691 ± 0.000 | 0.573 ± 0.004 | 0.892 ± 0.052 | 0.615 ± 0.009 |
| w/o LF | 0.952 ± 0.005 | 0.979 ± 0.006 | 0.994 ± 0.003 | 0.823 ± 0.027 | 0.775 ± 0.024 | 0.669 ± 0.029 | 0.841 ± 0.011 | 0.681 ± 0.005 | 0.608 ± 0.016 | 0.774 ± 0.045 | 0.607 ± 0.013 |
| w/o HF&LF | 0.938 ± 0.023 | 0.979 ± 0.003 | 0.990 ± 0.004 | 0.815 ± 0.017 | 0.774 ± 0.000 | 0.656 ± 0.001 | 0.838 ± 0.011 | 0.679 ± 0.004 | 0.646 ± 0.001 | 0.878 ± 0.034 | 0.627 ± 0.005 |
| w/o CIH | 0.951 ± 0.011 | 0.986 ± 0.000 | 0.974 ± 0.012 | 0.832 ± 0.017 | 0.805 ± 0.011 | 0.662 ± 0.015 | 0.856 ± 0.008 | 0.693 ± 0.005 | **0.558 ± 0.027** | **0.654 ± 0.046** | 0.549 ± 0.017 |
| w/o DTW | 0.946 ± 0.006 | 0.982 ± 0.004 | 0.966 ± 0.024 | 0.844 ± 0.012 | 0.810 ± 0.019 | 0.659 ± 0.014 | 0.857 ± 0.014 | 0.690 ± 0.007 | 0.596 ± 0.018 | 0.722 ± 0.002 | 0.580 ± 0.010 |
| w/o MA | 0.936 ± 0.021 | 0.970 ± 0.014 | 0.883 ± 0.030 | 0.794 ± 0.006 | 0.717 ± 0.038 | 0.654 ± 0.004 | 0.811 ± 0.007 | 0.683 ± 0.012 | 0.626 ± 0.042 | 1.158 ± 0.249 | 0.677 ± 0.015 |
| w/o CL | 0.948 ± 0.007 | 0.984 ± 0.000 | 0.988 ± 0.005 | 0.854 ± 0.001 | 0.811 ± 0.020 | 0.662 ± 0.010 | 0.853 ± 0.018 | 0.690 ± 0.009 | 0.577 ± 0.014 | 0.794 ± 0.093 | 0.574 ± 0.012 |
| w/o PT | 0.945 ± 0.009 | 0.982 ± 0.000 | 0.988 ± 0.012 | 0.844 ± 0.025 | 0.785 ± 0.040 | 0.667 ± 0.024 | 0.851 ± 0.020 | 0.695 ± 0.014 | 0.578 ± 0.001 | 0.840 ± 0.066 | **0.543 ± 0.006** |
| PrismNet | **0.953 ± 0.001** | **0.987 ± 0.000** | 0.996 ± 0.003 | **0.856 ± 0.002** | **0.825 ± 0.003** | **0.691 ± 0.002** | **0.867 ± 0.014** | **0.714 ± 0.001** | **0.558 ± 0.027** | **0.654 ± 0.046** | 0.549 ± 0.017 |


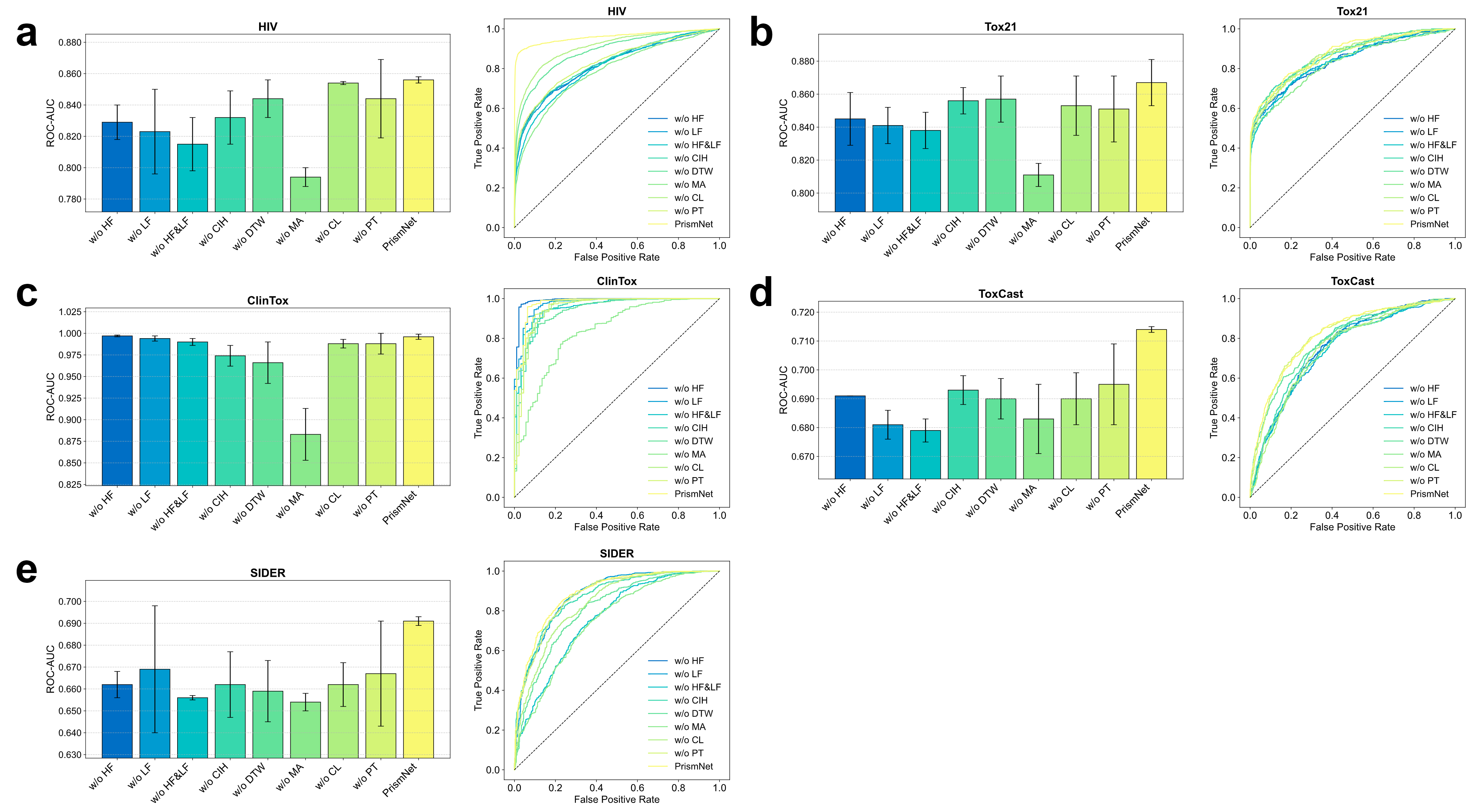


**Supplementary Figure 3.** Additional ablation results on several supplementary classification datasets further confirm that each core component of the PrismNet model is critical for maintaining its high performance. This figure, provided as a complement to the ablation analysis in the main text (Figure 5), presents detailed ablation study outcomes on five additional classification datasets. In each experiment, multiple model variants were created by systematically removing one key module at a time from the full PrismNet model and comparing their performance against the complete version. Each dataset is accompanied by a bar chart and a corresponding ROC curve plot. Error bars indicate standard deviation across three independent runs.

Specifically for the Lipophilicity dataset (as shown in Figure 6f and Table S7), we observed that the variant without pre-training (w/o PT) achieved a mean RMSE of 0.543, which is marginally lower than the Full PrismNet model's RMSE of 0.549. We attribute this observation primarily to a distribution shift between the pre-training and target domains. While the ZINC15 pre-training corpus covers a broad, generic chemical space, the Lipophilicity dataset occupies a specific niche focused on medicinal chemistry optimization sequences. In this specific context, the general topological priors learned from ZINC15 may not align perfectly with the target task, resulting in a mild form of negative transfer. However, critically, this numerical difference of 0.006 RMSE units is significantly smaller than the standard deviation of the full model. This indicates that the gap is not statistically significant and falls well within the expected range of stochastic fluctuation, demonstrating that PrismNet remains robust and avoids catastrophic performance degradation even in the presence of domain shifts.

**Hyperparameter Sensitivity**

**
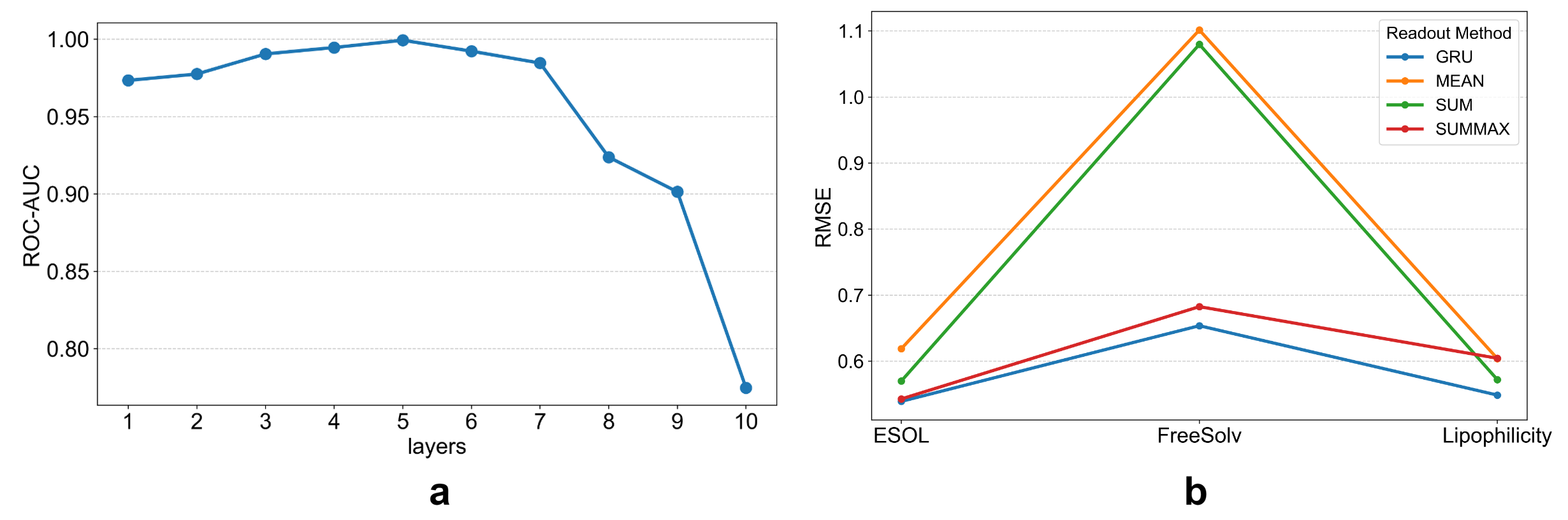
**

**Supplementary Figure 4.** Hyperparameter sensitivity analysis. Two sets of experiments were conducted to analyze how two key hyperparameters affect the performance of PrismNet. In each experiment, only one hyperparameter was varied while all others were held constant. The vertical axis indicates the model's performance metric on the specified task. **a** Investigation of how the number of GNN layers affects predictive accuracy. **b** Evaluation of four different readout functions across three regression datasets to assess their influence on model performance.

To assess the model's sensitivity to key hyperparameters, we conducted experiments on the ClinTox datasets and three regression benchmarks, focusing on how the number of GNN encoder layers $L$ and the choice of readout aggregation strategies affect model performance. All experiments were performed using identical data splits and training configurations. The results are presented in Figure S4.

For the number of layers in the GNN encoder $L$, the ROC-AUC exhibits a non-monotonic trend as $L$ increases from 1 to 10. When $L=1$, the model already achieves approximately 0.973 in ROC-AUC. As the depth increases, performance continues to improve, reaching a peak ROC-AUC of 0.99 at $L=5$. Beyond this point, performance begins to decline. At 7 layers, the score remains above 0.98, but noticeable degradation occurs from $L=8$ onward, dropping to around 0.90 at $L=9$ and plummeting to 0.78 at $L=10$. This pattern suggests that moderate network depth facilitates learning richer high-order structural features, whereas excessive depth leads to oversmoothing and gradient vanishing, ultimately reducing the model's discriminative capacity. Therefore, we select $L=5$ as the default number of encoder layers.

We compared four different readout aggregation strategies on three regression datasets: Mean, which computes the average of all node features; Sum, which performs direct summation; SumMax, which multiplies summed and max-pooled features element-wise; and GRU-based dynamic aggregation, which uses a bidirectional GRU to model inter-node dependencies. As shown in Figure S4b, the GRU-based readout consistently achieves superior performance across all tasks by more effectively integrating node features and capturing intra-molecular dependencies.

**Analysis of Dynamic Task Weighting in Multi-Task Learning**


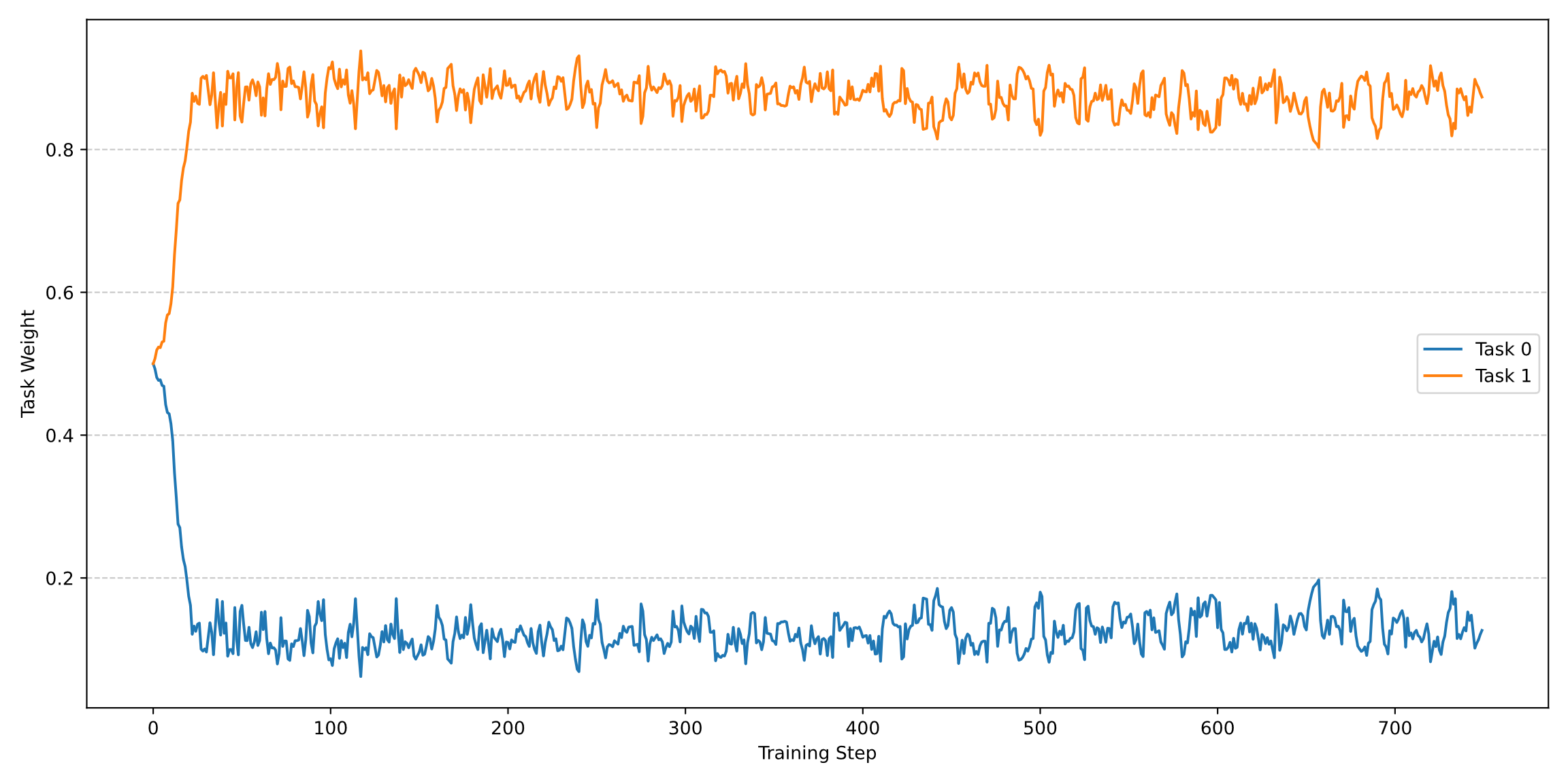


**Supplementary Figure 5.** Weight trajectories of the two tasks—FDA_APPROVED (Task 0) and CT_TOX (Task 1)—during training on the ClinTox dataset with a dynamic task weighting mechanism.

**Figure S5** illustrates the evolution of task weights during training on the ClinTox dataset when a dynamic task weighting mechanism is applied. The two tasks—FDA_APPROVED (Task 0) and CT_TOX (Task 1)—demonstrate distinct weight adjustment trends. This mechanism adaptively updates each task's weight based on the relative deviation between its current loss and historical average loss, thereby directing the model to focus more on tasks that converge slowly or are more challenging to learn.

As shown in the figure, the weight for CT_TOX rapidly rises to dominance and remains high throughout training, while the weight for FDA_APPROVED quickly drops and stabilizes at a lower level. From a pharmacological and biochemical perspective, this shift in attention is well-justified. The CT_TOX task involves predicting the cytotoxicity of compounds, a biologically complex property influenced by various mechanisms, such as genotoxicity caused by metabolic activation of aromatic amines, immune responses triggered by specific substituents, or covalent binding of electrophilic groups to proteins leading to cell damage. These structure–toxicity relationships are often highly localized, nonlinear, and structurally diverse, making them substantially more difficult to model.

In contrast, compounds labeled as FDA_APPROVED tend to share well-validated molecular scaffolds and physicochemical features, resulting in more consistent chemical patterns. This makes the task easier for the model to learn, as it can quickly capture these regularities. Under the guidance of the dynamic weighting strategy, the model naturally allocates more learning capacity to the biochemically more demanding CT_TOX task, demonstrating the effectiveness and rationality of the mechanism in multi-task learning.

**Attention Visualization Examples on Activity Cliff Pairs**


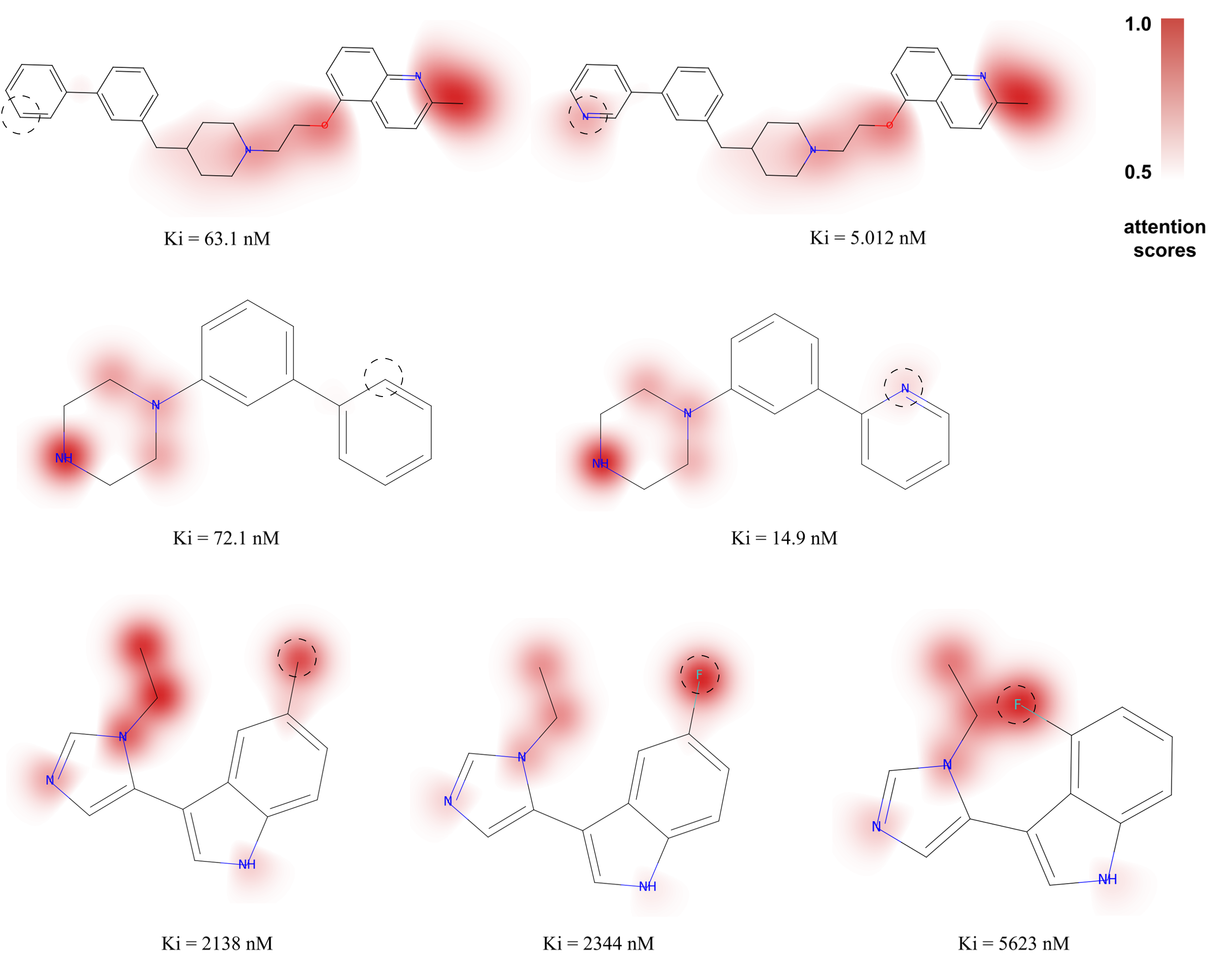


**Supplementary Figure 6.** Attention visualization examples for the PrismNet model on activity cliff pairs. The figure shows several pairs of molecules that exhibit high structural similarity yet show significant differences in bioactivity, as measured by the inhibition constant (Ki). Each pair differs only by subtle chemical modifications, which are indicated with dashed circles. The red shading denotes atom-level attention scores, where deeper color intensity reflects greater model focus and higher contribution of the corresponding atom to the final activity prediction.

**References**

1. Wu, Z., et al. MoleculeNet: a benchmark for molecular machine learning. *Chem. Sci.* **9,** 513-530 (2018).
2. Stanley, M., et al. Fs-mol: A few-shot learning dataset of molecules. *Thirty-fifth Conference on Neural Information Processing Systems Datasets and Benchmarks Track* (Round 2) (2021).
3. Huang, K., et al. Therapeutics data commons: Machine learning datasets and tasks for drug discovery and development. Preprint at <https://arxiv.org/pdf/2102.09548> (2021).
4. Zhou, G., et al. Uni-mol: A universal 3d molecular representation learning framework. Preprint at <https://doi.org/10.26434/chemrxiv-2022-jjm0j-v4> (2023).
5. Liu, S., Wang, H., Liu, W., Lasenby, J., Guo, H., & Tang, J. Pre-training molecular graph representation with 3d geometry. Preprint at <https://arxiv.org/pdf/2110.07728> (2021).
6. Stärk, H., et al. 3d infomax improves gnns for molecular property prediction. *International Conference on Machine Learning*. 20479-20502 (2022).
7. Rogers, D., & Hahn, M. Extended-connectivity fingerprints. *J. Chem. Inf. Model.* **50,** 742-754 (2010).
8. Xie, L., Xu, L., Kong, R., Chang, S., & Xu, X. Improvement of prediction performance with conjoint molecular fingerprint in deep learning. *Front. Pharmacol.* **11,** 606668 (2020).
9. Paszke, A., et al. Pytorch: An imperative style, high-performance deep learning library. *Advances in neural information processing systems* **32,** (2019).
10. Landrum, G. Rdkit: Open-source cheminformatics software. (2016).
11. Kingma, D. P., & Ba, J. Adam: A method for stochastic optimization. Preprint at <https://arxiv.org/pdf/1412.6980> (2014).
12. Sterling, T., & Irwin, J. J. ZINC 15–ligand discovery for everyone. *J. Chem. Inf. Model.* **55,** 2324-2337 (2015).
13. Subramanian, G., Ramsundar, B., Pande, V., & Denny, R. A. Computational modeling of β-secretase 1 (BACE-1) inhibitors using ligand based approaches. *J. Chem. Inf. Model.* **56,** 1936-1949 (2016).
14. Martins, I. F., Teixeira, A. L., Pinheiro, L., & Falcao, A. O. A Bayesian approach to in silico blood-brain barrier penetration modeling. *J. Chem. Inf. Model.* **52,** 1686-1697 (2012).
15. Gayvert, K. M., Madhukar, N. S., & Elemento, O. A data-driven approach to predicting successes and failures of clinical trials. *Cell Chem. Biol.* **23,** 1294-1301 (2016).
16. Artemov, A. V., Putin, E., Vanhaelen, Q., Aliper, A., Ozerov, I. V., & Zhavoronkov, A. Integrated deep learned transcriptomic and structure-based predictor of clinical trials outcomes. Preprint at <https://www.biorxiv.org/content/10.1101/095653v2> (2016).
17. Rohrer, S. G., & Baumann, K. Maximum unbiased validation (MUV) data sets for virtual screening based on PubChem bioactivity data. *J. Chem. Inf. Model.* **49,** 169-184 (2009).
18. Kuhn, M., Letunic, I., Jensen, L. J., & Bork, P. The SIDER database of drugs and side effects. *Nucleic Acids Res.* **44,** D1075-D1079 (2016).
19. Altae-Tran, H., Ramsundar, B., Pappu, A. S., & Pande, V. Low data drug discovery with one-shot learning. *ACS Cent. Sci.* **3,** 283-293 (2017).
20. Huang, R., et al. Tox21Challenge to build predictive models of nuclear receptor and stress response pathways as mediated by exposure to environmental chemicals and drugs. *Front. Environ. Sci.* **3,** 85 (2016).
21. Richard, Ann M., et al. ToxCast chemical landscape: paving the road to 21st century toxicology. *Chem. Res. Toxicol.* **29,** 1225-1251 (2016).
22. Delaney, J. S. ESOL: estimating aqueous solubility directly from molecular structure. *J. Chem. Inf. Comput. Sci.* **44,** 1000-1005 (2004).
23. Mobley, D. L., & Guthrie, J. P. FreeSolv: a database of experimental and calculated hydration free energies, with input files. *J. Comput. Aided Mol. Des.* **28,** 711-720 (2014).
24. Gaulton, A., et al. ChEMBL: a large-scale bioactivity database for drug discovery. *Nucleic Acids Res.* **40,** D1100-D1107 (2012).
25. Huang, K., Fu, T., Glass, L. M., Zitnik, M., Xiao, C., & Sun, J. DeepPurpose: a deep learning library for drug–target interaction prediction. *Bioinformatics* **36,** 5545-5547 (2020).
26. Kipf, T. N. Semi-supervised classification with graph convolutional networks. Preprint at <https://arxiv.org/pdf/1609.02907> (2016).
27. Hu, W., et al. Strategies for pre-training graph neural networks. Preprint at <https://arxiv.org/pdf/1905.12265> (2019).
28. Lee, W. H., Millman, S., Desai, N., Srivatsa, M., & Liu, C. Neuralfp: out-of-distribution detection using fingerprints of neural networks. *2020 25th International Conference on Pattern Recognition (ICPR)* 9561-9568 (2021).
29. Zhang, X., et al. Pushing the boundaries of molecular property prediction for drug discovery with multitask learning BERT enhanced by SMILES enumeration. *Research* **2022,** 0004 (2022).
30. Yang, K., et al. Analyzing learned molecular representations for property prediction. *J. Chem. Inf. Model.* **59,** 3370-3388 (2019).
31. Boral, N., Ghosh, P., Goswami, A., & Bhattacharyya, M. Accountable prediction of drug ADMET Properties with molecular descriptors. Preprint at <https://www.biorxiv.org/content/10.1101/2022.06.29.115436v1> (2022).
32. Huang, D., et al. A Unified System for Molecular Property Predictions: Oloren ChemEngine and its Applications. (2022).
33. Turon, G., Hlozek, J., Woodland, J. G., Kumar, A., Chibale, K., & Duran-Frigola, M. First fully-automated AI/ML virtual screening cascade implemented at a drug discovery centre in Africa. *Nat. Commun.* **14,** 5736 (2023).
34. Liu, S., Demirel, M. F., & Liang, Y. N-gram graph: Simple unsupervised representation for graphs, with applications to molecules. *Advances in neural information processing systems* **32,** (2019).
35. Rong, Y., et al. Self-supervised graph transformer on large-scale molecular data. *Advances in neural information processing systems* **33,** 12559-12571 (2020).
36. Wang, Y., Wang, J., Cao, Z., & Barati Farimani, A. Molecular contrastive learning of representations via graph neural networks. *Nat. Mach. Intell.* **4,** 279-287 (2022).
37. Zheng, K., et al. ESM all-atom: multi-scale protein language model for unified molecular modeling. Preprint at <https://arxiv.org/pdf/2403.12995> (2024).
38. Yang, J., et al. Mol-AE: auto-encoder based molecular representation learning with 3D cloze test objective. Preprint at <https://www.biorxiv.org/content/10.1101/2024.04.13.589331v2> (2024).
39. Xu, K., Hu, W., Leskovec, J., & Jegelka, S. How powerful are graph neural networks?. Preprint at <https://arxiv.org/pdf/1810.00826> (2018).
40. Gilmer, J., Schoenholz, S. S., Riley, P. F., Vinyals, O., & Dahl, G. E. Neural message passing for quantum chemistry. *International conference on machine learning* 1263-1272 (2017).
41. Fang, X., et al. Geometry-enhanced molecular representation learning for property prediction. *Nat. Mach. Intell.* **4,** 127-134 (2022).
